# Efficacy and Safety of Neuroendoscopy versus Craniotomy for Spontaneous Supratentorial Intracerebral Hemorrhage: An Updated Meta‐Analysis of Randomized and Non‐Randomized Studies

**DOI:** 10.1002/brb3.70581

**Published:** 2025-09-01

**Authors:** Muhammad Hassan Waseem, Zain ul Abideen, Nohela Rehman, Muhammad Haris Khan, Muhammad Fawad Tahir, Hafsa Arshad Azam Raja, Ameer Haider Cheema, Sania Aimen, Javed Iqbal

**Affiliations:** ^1^ Allama Iqbal Medical College Lahore Pakistan; ^2^ King Edward Medical University Lahore Pakistan; ^3^ Dow University of Health Sciences Karachi Pakistan; ^4^ Saidu Medical College Swat Pakistan; ^5^ H.B.S Medical and Dental College Islamabad Pakistan; ^6^ Rawalpindi Medical University Rawalpindi Pakistan; ^7^ UT Southwestern Medical Center Dallas Texas USA; ^8^ Quetta Institute of Medical Sciences Quetta Pakistan; ^9^ Hamad Medical Corporation Doha Qatar

**Keywords:** craniotomy, intracerebral hemorrhage, meta‐analysis, neuroendoscopy, supratentorial

## Abstract

**Background:**

Spontaneous supratentorial intracerebral hemorrhage (ICH) is a critical condition with high morbidity and mortality rates warranting urgent surgical evacuation. This systematic review and meta‐analysis compare the safety and efficacy of neuro‐endoscopy (NE) versus traditional craniotomy (CR) for managing ICH.

**Methods:**

From inception until July 2024, a comprehensive literature search was undertaken on PubMed, Cochrane Central, ScienceDirect, and Clinicaltrials.gov. Risk ratios (RR) and weighted mean differences (WMD) were pooled for categorical and continuous outcomes under the random effects model in Review Manager software. 5.4.1. Leave‐one‐out sensitivity analysis, Egger's regression, GRADE assessment, and meta‐regression were performed to evaluate the heterogeneity, publication bias, certainty of evidence, and effect size variability, respectively.

**Results:**

Our meta‐analysis included eight clinical trials and 20 cohort studies with 9,437 patients. NE outperformed CR in terms of favorable neurological outcomes (RR = 1.59, 95% CI: [1.30,1.94]; p < 0.00001; I^2^ = 90%), mortality (RR = 0.62, 95% CI:[0.48,0.81]; p < 0.0004; I^2^ = 51%), hematoma evacuation rate (WMD = 7.17, 95% CI: [4.68,9.65]%; p < 0.00001; I ^2^ = 94%) operating time (WMD = ‐102.08 min, 95 CI: [‐120.29,‐83.87]; p < 0.00001; I^2^ = 98%), blood loss (WMD = ‐255.51 mL, 95% CI: [‐383.61,‐127.41]; p < 0.0001; I^2^ = 100%), length of hospital stay (WMD = ‐3.34 days, 95% CI: [‐5.05,‐1.64] days; p < 0.0001; I^2^ = 62%), ICU stay (WMD = ‐2.85, 95% CI: [‐5.13,‐0.57] days; p < 0.01; I^2^ = 96%), meningitis (RR = 0.58, 95% CI: [0.36,0.95]; p < 0.03; I^2^ = 10 %), infections (RR = 0.49, 95% CI: [0.35,0.67]; p < 0.0001; I^2^ = 51%), residual hematoma volume (MD = ‐2.22; 95% CI: [‐3.37,‐1.07]; p < 0.0002; I^2^ = 90%) and overall complications (RR = 0.52, 95% CI: [0.40‐0.67]; p < 0.00001; I^2^ = 70%). In addition, re‐bleeding was found to be comparable between the CR and NE groups (p = 0.08).

**Conclusion:**

NE treatment for spontaneous intracerebral hemorrhage (ICH) is associated with favorable neurological outcomes, decreased mortality, shorter operating time, reduced blood loss and residual volume, shorter length of hospital and intensive care unit (ICU) stay, and fewer infections, meningitis, and overall complications; however, the re‐bleeding rate was found to be comparable.

## Graphical abstract

Neuroendoscopic treatment for spontaneous intracerebral hemorrhage (ICH) results in better neurological outcomes, lower mortality, reduced surgery time, less blood loss, and shorter hospital and ICU stays compared to craniotomy. The updated meta‐analysis highlights neuroendoscopy as a more effective and efficient approach for managing spontaneous ICH.

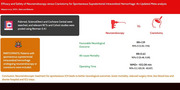

## 1 Introduction

Supratentorial intracerebral hemorrhage (ICH) is a severe and life‐threatening condition characterized by bleeding within the brain's supratentorial compartments, often resulting in significant morbidity and mortality (Rajashekar and Liang 2023; Nagasaka et al. 2011). This type of hemorrhage accounts for approximately 10–15% of all strokes. The case fatality rate for ICH is notably high, with 40% of patients dying within one month and 54% in one year. Among those who survive, only 12% to 39% can attain long‐term functional independence (An et al. 2017). Conventional management strategies for supratentorial ICH typically involve surgical interventions to alleviate intracranial pressure and remove hematomas. Traditional treatments include craniotomy (CR), which involves the removal of a portion of the skull to access and evacuate the hemorrhage, and stereotactic aspiration, which utilizes a minimally invasive approach to aspirate the blood clot (Wilting et al. 2022).

Multiple randomized controlled trials (RCTs) and meta‐analyses have examined the effectiveness of surgical interventions for supratentorial ICH (Mendelow et al. 2013; Vespa et al. 2016; Ye et al. 2017; Liang et al. 2017). Although surgery is a common approach for treating hypertensive cerebral hemorrhage, outcomes can vary depending on the specific surgical technique used (Liang et al. 2017; Zhao and Zhou 2016). Neuroendoscopy (NE) involves the use of an endoscope to access the hemorrhage through small burr holes or keyhole incisions, offering the advantage of reduced tissue disruption, shorter recovery times, and fewer complications such as infections and postoperative pain (Sun et al. 2019). Advances in minimally invasive techniques have led to alternatives to traditional CR, including NE surgery. NE surgery is designed to remove clots while minimizing damage to surrounding brain tissue more effectively (Nagasaka et al. 2011). Conversely, CR offers direct access to the hemorrhage, facilitating thorough clot removal and potentially better bleeding control. However, CR is associated with longer operative times, increased surgical trauma, and a higher risk of postoperative complications (Kellner et al. 2021).

Despite the growing body of literature on these techniques, the results from various studies have shown inconsistent findings regarding their relative efficacy and safety. A previous meta‐analysis of RCTs evaluated the efficacy and safety of NE versus CR, which demonstrated that NE was linked to more favorable functional outcomes and reduced rates of functional disabilities compared to CR (Monteiro G. de et al. 2024). Recent multicenter trials and large‐scale studies have emerged, providing new insights and data that could impact the current understanding of these surgical approaches. Therefore, an updated meta‐analysis is warranted to re‐evaluate and compare the efficacy and safety of NE versus CR in managing spontaneous supratentorial ICH. This study aims to address this gap in the literature by synthesizing recent evidence to determine which surgical technique offers superior outcomes in terms of both efficacy and safety.

## 2 Methods

We conducted a systematic review and meta‐analysis following the guidelines of the Preferred Reporting Items for Systematic Reviews and Meta‐Analyses (PRISMA) (Page et al. 2021) and the *Cochrane Handbook for Systematic Reviews of Interventions* (Higgins et al. 2019). This review's protocol was pre‐registered on PROSPERO under the ID: **CRD42024576501**.

### 2.1 Search Strategy

From their inception until August 2024, PubMed, Cochrane Central, ScienceDirect, and Clinicaltrials.gov databases were comprehensively searched. The search strategy utilized the following MeSH terms and keywords: “Neuroendoscopy,” “Intracranial Hemorrhages” and “Craniotomy.” Relevant articles were located by manually searching bibliographies. A detailed description of the search strategy for each electronic database is reported in Supplementary Table S1.

### 2.2 Study Selection and Eligibility Criteria

**FIGURE 1:**
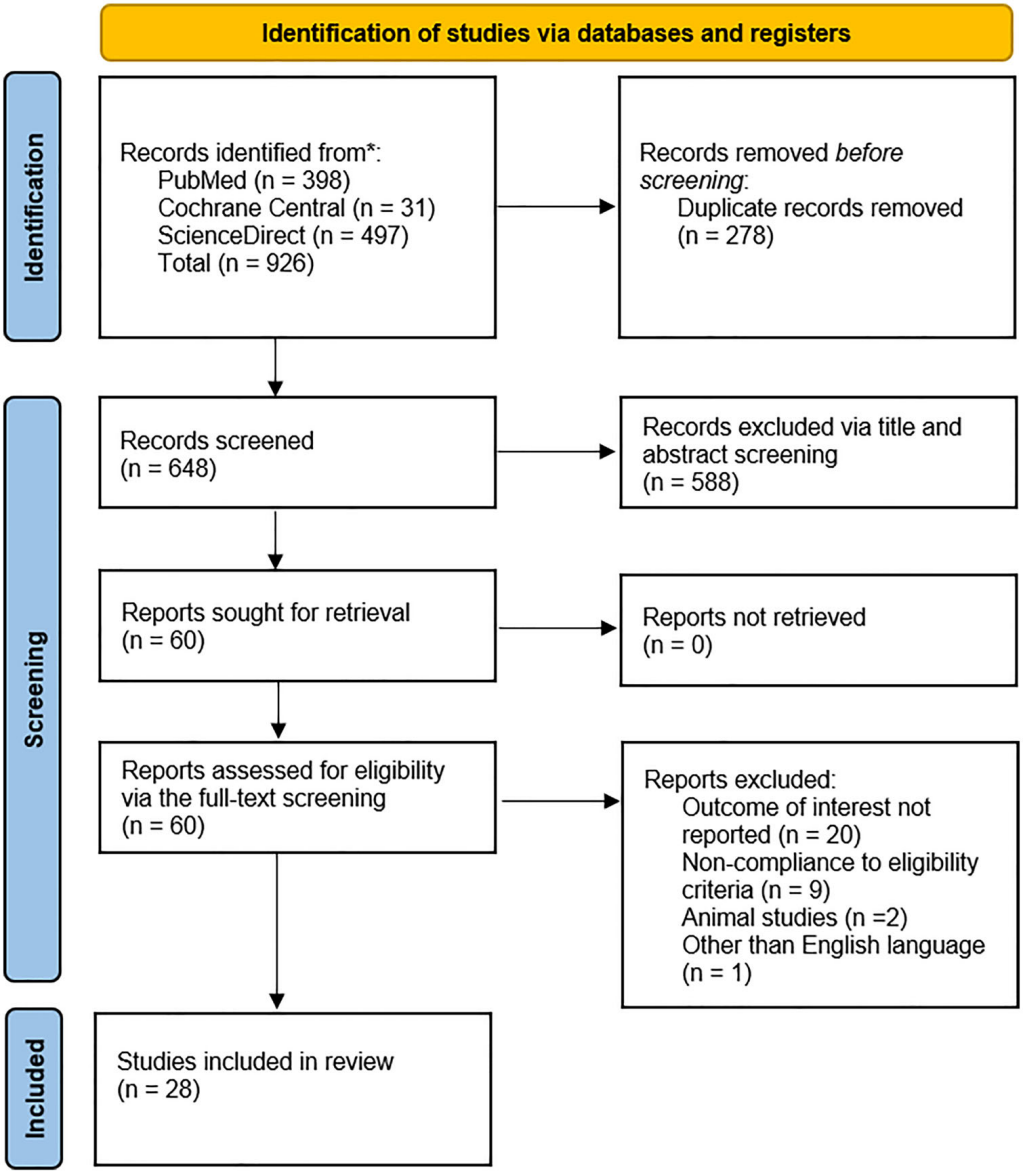
PRISMA flowchart of the study selection process.

All articles found in our search were imported into EndNote version 20, and any duplicates were removed. Two authors (NR and HAAR) independently reviewed the articles' titles and abstracts, excluding those that did not meet the eligibility criteria. The full texts of the remaining articles were examined to assess their eligibility against the predetermined criteria. Conflicts or disagreements were addressed and resolved with the involvement of a third author (MHW). The eligibility criteria for inclusion were patients aged > 18 years with spontaneous supratentorial intracerebral hemorrhage (ICH) who had undergone hematoma evacuation either via neuroendoscopy (NE) or craniotomy. Patients aged less than 18 years, along with traumatic or infratentorial ICH undergoing stereotactic aspiration, were excluded. Regarding study designs, RCTs and cohorts are to be included, while case reports, case series, reviews, and protocols are excluded. The detailed selection process is depicted in the PRISMA flowchart in Figure 1.

### 2.3 Data Extraction

A predefined Microsoft Excel spreadsheet was employed for data extraction by two authors (ZUA and HK). Any discrepancies were resolved by a third author (MHW). Data on study characteristics (author name, publication year, study location, number of participants, institution or database used, and study design), patient characteristics (age, sex, ICH volume, mean preoperative Glasgow Coma Scale (GCS), time to surgery, intracerebral hematoma location), and follow‐up were extracted. The primary outcome of interest was favorable neurological function. The secondary outcomes were mortality, hematoma evacuation rate, operating time, blood loss, length of hospital stay, ICU stay, rebleeding, overall complications, meningitis, infections, and residual hematoma volume. A favorable neurological outcome is defined as a Glasgow Prognosis Scale (GPS) grade IV or V, a Glasgow Outcome Scale (GOS) score of 4–5 points, an Activity of Daily Living (ADL) score of 1–3 points, or a modified Rankin scale (mRS) score of 0–3 points. Note that the RCT by Gui et al. has taken the ADL score of 4–5 points as indicative of a favorable neurological prognosis.

### 2.4 Outcome Definitions

The GPS is a prognostic tool used to predict the potential for recovery in patients with neurological conditions. The scale classifies recovery into five grades (Grades I‐V), with Grades IV and V indicating favorable neurological outcomes. The ADL scale is used to assess the level of independence or dependence a patient has in performing daily activities, such as eating, bathing, dressing, grooming, and mobility. In this study, ADL grades I to III are associated with a good prognosis, indicating that patients are either independent or require minimal assistance in daily activities. These grades represent patients who can maintain a relatively high level of functional independence after treatment. On the other hand, ADL grades IV and V indicate a poor prognosis, with patients needing significant assistance or being completely dependent on caregivers for basic daily functions. The modified Rankin Scale (mRS) is a 7‐point scale used to assess the degree of disability or dependence in daily activities, with a score of 0 indicating no disability and scores ranging from 1 to 6 indicating increasing levels of disability. In this study, we have defined favorable neurological outcomes as an mRS score of 0–3 points.

### 2.5 Risk of Bias

The Cochrane Risk of Bias tool for RCTs (RoB 2.0) (Sterne et al. 2019) was employed by two authors (NR and HAAR) to evaluate the quality of the included RCTs, whereas the Newcastle‐Ottawa Scale (NOS) (Ga 2013) was used to evaluate the included observational cohorts. In the event of any disagreements, a third author was consulted to reach an agreement. RoB 2.0 is organized into five domains. (1) bias arising from the randomization process, (2) bias due to deviations from intended interventions, (3) bias due to missing outcome data, (4) bias in the measurement of the outcome, and (5) bias in the selection of the reported result, whereas the NOS judges the studies based on the three domains: selection, comparability, and outcome. The publication bias of the included studies was assessed visually through funnel plots and statistically through Egger's regression test. To determine the certainty of evidence, we performed the GRADE assessment of all the extracted outcomes through the GRADEpro GDT (GRADEpro n.d.).

### 2.6 Data Analysis

We pooled categorical data as risk ratios (RR) with 95% confidence intervals (95% CI) and pooled continuous data as mean differences (MD) with 95% CI. We employed the Mantel‐Haenszel random effects model and the inverse variance random effects model to pool RRs and MDs, respectively. If the data was provided in the form of median, range, or interquartile range (IQR), we calculated the mean and standard deviation using the methods described by Wan et al. and Luo et al. Subgroup analysis was done based on the RCT or cohort study design. Heterogeneity was evaluated using Higgins I^2^ statistics (Higgins et al. 2003) and the Cochrane Q test, with p < 0.05 considered statistically significant. With outcomes showing heterogeneity of more than 50%, we further evaluated the cause of heterogeneity using a leave‐one‐out sensitivity analysis. Meta‐regression with the Omnibus test was performed using the Open meta‐analyst software (OpenMeta[Analyst]–CEBM @ Brown n.d.) to statistically analyze the confounding effect of continuous baseline variables on outcomes via linear regression. Forest plots were generated via the Review Manager (RevMan, Version 5.4.1; The Cochrane Collaboration, Copenhagen, Denmark) software (RevMan n.d.).

## 3 Results

### 3.1 Search Results

A detailed search across various electronic databases, including PubMed, Cochrane Library, and ScienceDirect, yielded a total of 926 articles, with the following distribution: PubMed (398), Cochrane Library (31), and ScienceDirect (497). After removing duplicates (n = 278), we were left with 648 records. The obtained studies were passed through title and abstract screening, yielding a total of 60 articles to be included in full‐text screening, after which only 28 (Sun et al. 2019; Gui et al. 2019; Cho et al. 2006; Zhang et al. 2014; Feng et al. 2016; Zhang et al. 2018; Qiu et al. 2003; Zhu et al. 2012; Chi et al. 2014; Wang et al. 2015; Yamashiro et al. 2015; Cai et al. 2017; Li et al. 2017; Xu et al. 2017; Eroglu et al. 2018; Fu et al. 2019; Noiphithak et al. 2023; Xu et al. 2024; Li et al. 2022; He et al. 2023; Lv et al. 2023; Wang et al. 2024; Kondabathini et al. 2024; Katsuki et al. 2020; Tahara et al. 2023; Yang et al. 2024; Fujita et al. 2021; Du et al. 2021) studies were included in the final quantitative analysis.

### 3.2 Characteristics of Included Studies

This review includes eight RCTs (Gui et al. 2019; Cho et al. 2006; Feng et al. 2016; Zhang et al. 2018; Noiphithak et al. 2023; Xu et al. 2024; Lv et al. 2023; Zhang et al. 2014) and 20 cohorts (Sun et al. 2019; Qiu et al. 2003; Zhu et al. 2012; Chi et al. 2014; Wang et al. 2015; Yamashiro et al. 2015; Cai et al. 2017; Li et al. 2017; Xu et al. 2017; Eroglu et al. 2018; Fu et al. 2019; Li et al. 2022; He et al. 2023; Wang et al. 2024; Kondabathini et al. 2024; Katsuki et al. 2020; Tahara et al. 2023; Yang et al. 2024; Fujita et al. 2021; Du et al. 2021) published between 2003 and 2024. The studies encompassed 9,437 patients, with sample sizes ranging from 22 to 5,396. The mean age of the population ranged from 49 to 81 years. The mean ICH volume ranged from 30 to 132 mL, while the mean preoperative GCS ranged from 5 to 15. The maximum follow‐up duration was 21 months, with most cases of ICH being operated on within 24 h. Twenty‐one studies were conducted in China (Sun et al. 2019; Gui et al. 2019; Cho et al. 2006; Feng et al. 2016; Zhang et al. 2018; Qiu et al. 2003; Zhu et al. 2012; Chi et al. 2014; Wang et al. 2015; Cai et al. 2017; Li et al. 2017; Xu et al. 2017; Fu et al. 2019; Xu et al. 2024; Li et al. 2022; He et al. 2023; Lv et al. 2023; Wang et al. 2024; Yang et al. 2024; Du et al. 2021; Zhang et al. 2014), while the remaining studies were conducted in Japan (Yamashiro et al. 2015; Katsuki et al. 2020; Tahara et al. 2023; Fujita et al. 2021), Turkey (Eroglu et al. 2018), Thailand (Noiphithak et al. 2023), and India (Kondabathini et al. 2024). The details are provided in Table 1. The retrospective cohort study conducted by Chi et al. has stratified the CR into two groups, one with a standard bone flap and one with a small bone flap, also known as the keyhole approach. Therefore, we have conducted two comparisons in our quantitative synthesis: one comparing NE versus standard bone flap CR (Chi et al. [A]) and the other comparing NE versus keyhole CR (Chi et al. [B]).

**TABLE 1:** Baseline characteristics of the included studies.

Author year	Site	Study design	Sample size (n)	Gender (M/F)	Mean age in years (SD)		Mean ICH volume mL (SD)		Mean preoperative GCS score (SD)		Time to surgery (hr)	Follow‐up duration (months)
					NE	CR	NE	CR	NE	CR		
Cho et al. 2006	China	RCT	60	40/20	56.67 (8.66)	54.22 (10.47)	55.48 (23.25)	42.11(18.43)	9.26 (1.22)	9.32 (1.03)	<24	6
Zhang et al. 2014	China	RCT	51	38/13	59.90 (12.85)	61.45 (9.25)	58.28 (18.84)	62.16(15.62)	9.19 (3.76)	8.37(2.39)	<24	8
Feng et al. 2016	China	RCT	184	114/70	66.35(12.23)	69.1(10.26)	≤ 60	≤ 60	9.19 (3.76)	8.37 (2.39)	—	6
Zhang et al. 2018	China	RCT	130	70/60	62.4(2.8)	62.4(2.8)	39.1(6.2)	39.0(6.1)	—	—	—	3
Qiu et al. 2003	China	RC	47	25/22	62(8)	60(10)	30‐70	30‐70	6‐15	6‐15	<48	21
Zhu et al. 2011	China	RC	58	32/26	60.6(7.2)	64.6(5.0)	53.7(15.8)	63.9(17.0)	8(2)	7(2)	<24	3
Chi et al. 2014 (A)	China	RC	456	257/199	62.8(9.2)	63.4(8.62)	58.2(17.53)	83.4(27.51)	5‐15	5‐15	<24	6
Chi et al. 2014 (B)	China	RC	442	248/194	62.8(9.2)	62.7(9.30)	58.2(17.53)	56.3(19.31)	5‐15	5‐15	<24	6
Wang et al. 2015	China	RC	45	34/11	57.3	56.9	61.2	47.1	8	7	<6	12
Yamashir et al. 2015	Japan	RC	22	14/8	70.4(10.7)	58.2(24.0)	131.7(52.2)	99.2(16.5)	—	—	—	—
Cai et al. 2017	China	RC	41	25/16	59.6(10.1)	57.4(14.6)	51.7(19.6)	56.3(23.5)	7.6(2.7)	7.2(2.0)	—	6
Li et al. 2017	China	RC	63	34/29	60.7(8.8)	61.8(9.7)	54.5(14.2)	59.9(14.6)	8.6(3.0)	7.9(2.7)	<24	6
Xu et al. 2015	China	RC	164	114/50	52.3(12.0)	54.5(13.9)	56.0(31.4)	56.3(29.8)	7.7(2.1)	7.8(3.0)	<24	6
Eroglu et al. 2018	Turkey	RC	34	22/12	56.23(12.73)	53.64(12.38)	53.07(4.64)	51.47(4.14)	6	5	<24	6
Fu et al. 2018	China	RC	121	58/63	61.6(9.2)	63.2(9.4)	49.8(11.3)	50.9(12.4)	8(2.9)	7.9(2.8)	<24	6
Gui et al. 2019	China	RCT	126	75/51	54.02(3.74)	52.33(3.41)	30‐60	30‐60	6.01(0.64)	6.35(0.71)	<12	3
Sun et al. 2018	China	RC	89	47/42	50.3 (7.1)	48.9 (6.5)	47.2 (5)	46.8 (7.6)	8.7 (2.9)	8.6 (3.1)	<24	6
Noiphithak et al. 2023	Thailand	RCT	188	130/58	51 (18)	50 (14)	50.1(33)	49.3 (28.9	10(4)	10(6)	<7	6
Xu et al. 2024	China	RCT	475	326/149	56.6 (11.0)	55.8 (11.8)	49.1 (20.3)	49.9 (17.6)	9.0 (3.1)	8.9 (3.0)	<18	6
Li et al. 2022	China	RC	106	39/33	67.08(6.79)	66.12 (6.35)	—	—	4.74 (1.46)	4.36 (1.23)	—	3
He et al. 2023	China	RC	126	96/30	53.97 (11.87)	54.44 (9.92)	—	—	8.09(1.52)	8.05(1.48)	<12	6
Lv et al. 2023	China	RCT	128	85/43	56.74(13.69)	54.76(12.62)	—	—	7.48(2.12)	6.96(2.49)	<13	6
Wang et al. 2024	China	RC	81	63/18	55.28(13.82)	53.67(12.57)	43.70(17.93)	46.99(22.51)	9.00(2.50)	8.42 (3.03)	—	6
Kondabathini et al. 2024	India	PC	45	34/11	50	50	—	—	—	—	—	6
Katsuki et al. 2020	Japan	RC	134	78/56	74.7648(12.8843)	70.5299 (15.9055)	102.2352 (55.3266)	128.4688 (74.2255)	10.353 (3.7895)	9.7057 (4.5444)	<24	6
Tahara et al. 2023	Japan	RC	5,396	2354/ 3042	81.2 (4.4)	81.2 (4.5)	—	—	—	—	<48	—
Yang et al. 2024	China	RC	193	136/57	56.5619(10.3629)	56.1522 (10.4576)	—	—	—	—	<24	6
Du et al. 2021	China	RC	360	234/126	76 (35.85)	51 (34.46)	—	—	—	—	—	6
Fujita et al. 2021	Japan	RC	72	45/27	73.2(6.4)	72.2(6.4)	63.3(26.1)	68.1(29.5)	10.3551 (3.8521)	9.5553 (3.6762)	—	—

***Abbreviations*: CR**, craniotomy; **GCS**, Glasgow Coma Scale; **ICH**, intracerebral hemorrhage; **NE**, neuroendoscopy; **PC**, prospective cohort; **RCT**, randomized controlled trial; **RC**, retrospective cohort.

### 3.3 Risk of Bias and GRADE Assessment

The NOS (Ga 2013) was used to assess the quality of observational cohort studies. One of the studies was of moderate quality, with a NOS score of 6 (Li et al. 2017), while the remaining studies were of high quality, with NOS scores of 7 (Sun et al. 2019; Qiu et al. 2003; Wang et al. 2015; Yamashiro et al. 2015; Li et al. 2017; He et al. 2023; Kondabathini et al. 2024; Katsuki et al. 2020; Tahara et al. 2023; Yang et al. 2024; Fujita et al. 2021; Du et al. 2021) and 8 (Zhu et al. 2012; Chi et al. 2014; Cai et al. 2017; Xu et al. 2017; Eroglu et al. 2018; Fu et al. 2019; Li et al. 2022; Wang et al. 2024). Details regarding the quality assessment of the observational studies were provided in Supplementary Table S2. The RoB 2 tool (Sterne et al. 2019) was used to evaluate the quality of the clinical trials. 1 of the studies reported a high risk of bias (Feng et al. 2016), whereas the remaining showed some concerns (Gui et al. 2019; Cho et al. 2006; Zhang et al. 2018; Lv et al. 2023) or low risk (Noiphithak et al. 2023; Xu et al. 2024; Zhang et al. 2014) of bias. The traffic plot for the quality assessment of the clinical trials is provided in Supplementary Figure S1. The GRADE assessment was utilized to evaluate the certainty of evidence, as depicted in Table 2.

**TABLE 2:** GRADE assessment for certainty of evidence.

Patient or population: Spontaneous supratentorial hemorrhage	Intervention: Neuroendoscopy	Comparison: Craniotomy			
Outcomes	Anticipated absolute effects^*^ (95% CI)		Relative effect (95% CI)	№ of participants (studies)	GRADE
	Risk with craniotomy	Risk with Neuroendoscopy			
favorable neurological outcome—Cohorts	417 per 1,000	**700 per 1,000** (517 to 946)	**RR 1.68** (1.24 to 2.27)	7411 (10 OS)	⨁◯◯◯ Very low^a^
favorable neurological outcome—RCTs	376 per 1,000	**533 per 1,000** (451 to 631)	**RR 1.42** (1.20 to 1.68)	1250 (7 RCTs)	⨁⨁⨁⨁ High
Mortality—Cohorts	152 per 1,000	**85 per 1,000** (59 to 120)	**RR 0.56** (0.39 to 0.79)	7855 (18 OS)	⨁⨁◯◯ Low
Mortality—RCTs	116 per 1,000	**105 per 1,000** (77 to 143)	**RR 0.90** (0.66 to 1.23)	1310 (8 RCTs)	⨁⨁⨁◯ Moderate^b^
Hematoma evacuation rate—Cohorts	The mean hematoma evacuation rate—Cohorts ranged from **69–96** mL	MD **6.86 mL higher** (2.91 higher to 10.82 higher)	—	999 (12 OS)	⨁◯◯◯ Very low^a^
Hematoma evacuation rate—RCTs	The mean hematoma evacuation rate—RCTs ranged from **73–94** mL	MD **7.52 mL higher** (3.73 higher to 11.3 higher)	—	1354 (8 RCTs)	⨁⨁⨁◯ Moderate^a^
Operating time—Cohorts	The mean operating time—Cohorts ranged from **63–306** min	MD **94.11 min lower** (114.45 lower to 73.77 lower)	—	1263 (15 OS)	⨁⨁◯◯ Low^a^
Operating time—RCTs	The mean operating time—RCTs ranged from **76–332** min	MD **118.49 min lower** (147.3 lower to 89.67 lower)	—	1154 (7 RCTs)	⨁⨁⨁◯ Moderate^a^
Blood loss (ml)—Cohorts	The mean blood loss (ml)—Cohorts ranged from **42–812** mL	MD **303.81 mL lower** (461.31 lower to 146.32 lower)	—	1067 (11 OS)	⨁⨁◯◯ Low^a^
Blood loss (ml)—RCTs	The mean blood loss (ml)—RCTs ranged from **36–277** mL	MD **152.95 mL lower** (261.68 lower to 44.22 lower)	—	1043 (5 RCTs)	⨁⨁⨁◯ Moderate^a^
Length of hospital stay—Cohorts	The mean length of hospital stay—Cohorts ranged from **10–45** days	MD **3.88 days lower** (5.25 lower to 2.52 lower)	—	805 (7 OS)	⨁⨁◯◯ Low
Length of hospital stay—RCTs	The mean length of hospital stay—RCTs ranged from **24–48** days	MD **3.36 days lower** (14.33 lower to 7.61 higher)	—	535 (2 RCTs)	⨁⨁◯◯ Low^a^, ^c^
ICU stay (days)—Cohorts	The mean ICU stay (days)—Cohorts ranged from **5–12** days	MD **3.35 days lower** (5.97 lower to 0.74 lower)	—	335 (4 OS)	⨁◯◯◯ Very low^a^
ICU stay (days)—RCTs	The mean ICU stay (days)—RCTs ranged from **6–14** days	MD **1.52 days lower** (3.68 lower to 0.64 higher)	—	663 (3 RCTs)	⨁⨁◯◯ Low^a^, ^c^
Rebleeding—Cohorts	67 per 1,000	**56 per 1,000** (34 to 90)	**RR 0.83** (0.51 to 1.35)	1208 (14 OS)	⨁◯◯◯ Very low^b^
Rebleeding—RCTs	58 per 1,000	**37 per 1,000** (19 to 71)	**RR 0.63** (0.33 to 1.22)	786 (4 RCTs)	⨁⨁⨁◯ Moderate^b^
Overall complications—Cohorts	79 per 1,000	**38 per 1,000** (28 to 53)	**RR 0.49** (0.36 to 0.68)	6604 (15 OS)	⨁⨁◯◯ Low
Overall complications—RCTs	432 per 1,000	**238 per 1,000** (151 to 372)	**RR 0.55** (0.35 to 0.86)	1224 (7 RCTs)	⨁⨁⨁◯ Moderate^a^
meningitis—Cohorts	15 per 1,000	**6 per 1,000** (3 to 14)	**RR 0.40** (0.18 to 0.90)	6086 (7 OS)	⨁⨁◯◯ Low
meningitis—RCTs	48 per 1,000	**36 per 1,000** (19 to 67)	**RR 0.74** (0.39 to 1.40)	989 (5 RCTs)	⨁⨁⨁◯ Moderate^b^
Infections—Cohorts	32 per 1,000	**13 per 1,000** (9 to 20)	**RR 0.42** (0.29 to 0.62)	6138 (9 OS)	⨁⨁◯◯ Low
Infections—RCTs	285 per 1,000	**163 per 1,000** (100 to 263)	**RR 0.57** (0.35 to 0.92)	1224 (7 RCTs)	⨁⨁⨁◯ Moderate^a^
Residual hematoma volume—Cohorts	The mean residual hematoma volume—Cohorts ranged from **4–23** mL	MD **3.94 mL lower** (9.1 lower to 1.22 higher)	—	153 (2 OS)	⨁◯◯◯ Very low^a^, ^c^
Residual hematoma volume—RCTs	The mean residual hematoma volume—RCTs ranged from **2–13** mL	MD **2.39 mL lower** (4.52 lower to 0.25 lower)	—	518 (4 RC+Ts)	⨁⨁⨁◯ Moderate^a^

***Abbreviations***: **CI**, confidence interval; **MD**, mean difference; **OS**, observation studies; **RR**, risk ratio; **RCTs**, randomized controlled trials.

^a^ Heterogeneity is more than 60%.

^b^ 95 % CI is wide and crossing the 1.

^c^ 95 % CI is wide and crossing the 0.

## 4 Outcomes

### 4.1 Favorable Neurological Outcome

**FIGURE 2:**
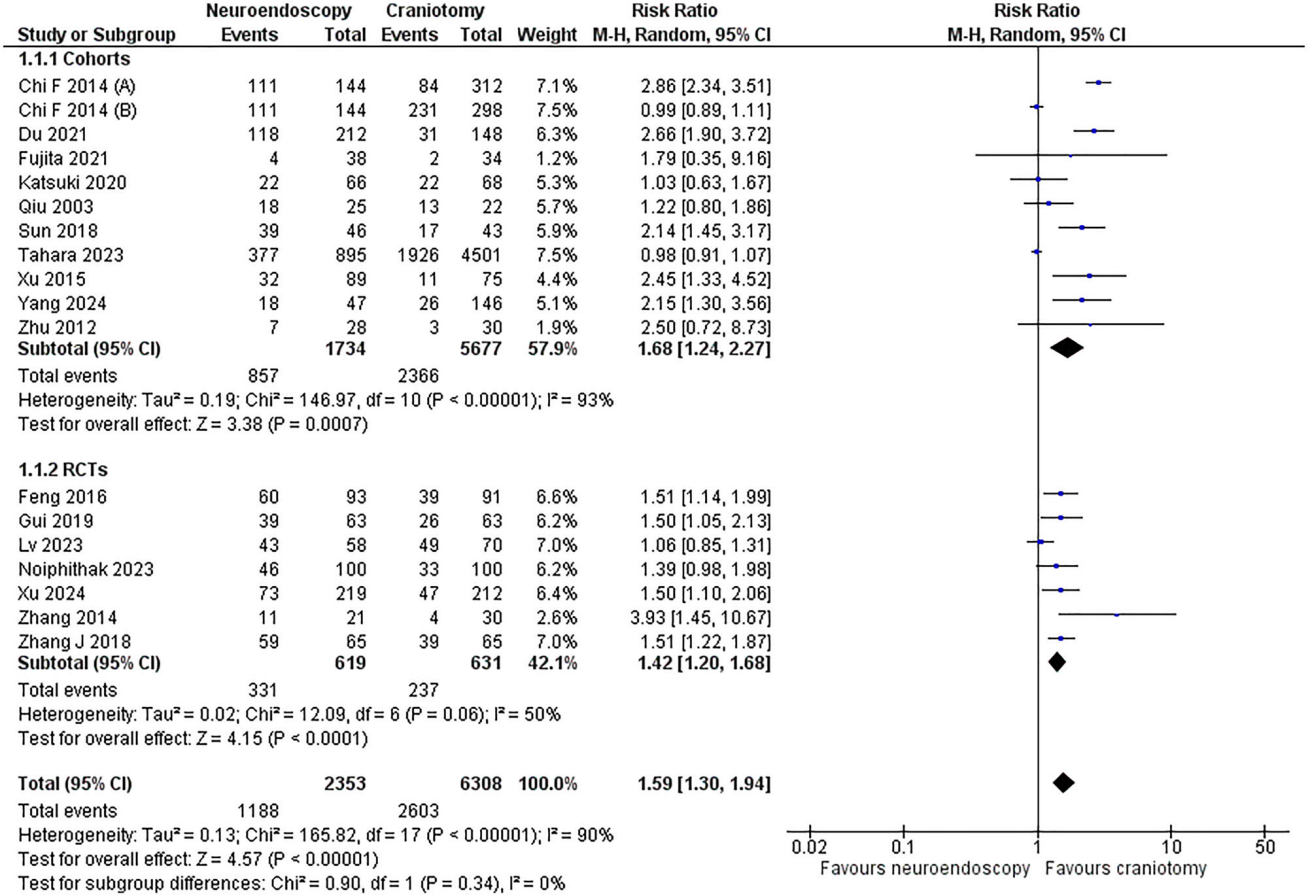
Favorable Neurological Outcome FOREST PLOT.

Seventeen studies (Sun et al. 2019; Gui et al. 2019; Feng et al. 2016; Zhang et al. 2018; Qiu et al. 2003; Zhu et al. 2012; Chi et al. 2014; Xu et al. 2017; Noiphithak et al. 2023; Xu et al. 2024; Lv et al. 2023; Katsuki et al. 2020; Tahara et al. 2023; Yang et al. 2024; Fujita et al. 2021; Du et al. 2021; Zhang et al. 2014) with 7 RCTs (Gui et al. 2019; Zhang et al. 2018; Noiphithak et al. 2023; Xu et al. 2024; Lv et al. 2023; Zhang et al. 2014; Feng et al. 2016) and 10 cohorts (Sun et al. 2019; Qiu et al. 2003; Zhu et al. 2012; Chi et al. 2014; Xu et al. 2017; Katsuki et al. 2020; Tahara et al. 2023; Yang et al. 2024; Fujita et al. 2021; Du et al. 2021) comprising 3,791 events: 2,353 patients in the NE group (27%) and 6,308 patients in the CR group(73%) reported the favorable neurological outcome. The NE approach was significantly superior to CR regarding this outcome (RR = 1.59, 95% CI: [1.30,1.94]; p < 0.00001). The heterogeneity was high (I^2^ = 90%; p < 0.00001). On subgroup analysis, the NE remained superior to the CR approach (Figure 2).

### 4.2 Mortality

**FIGURE 3:**
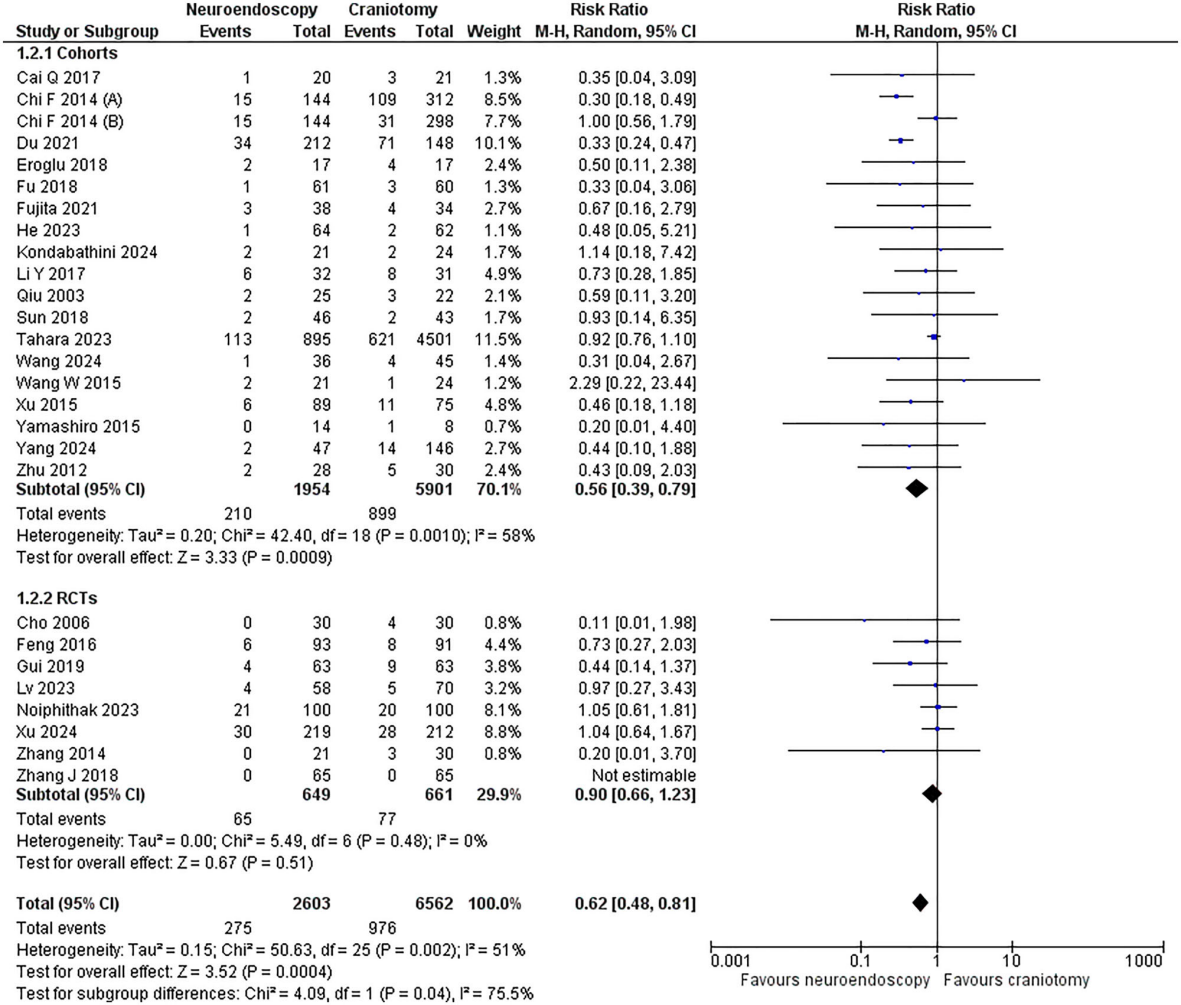
Mortality Forest Plot.

A total of 26 studies (Sun et al. 2019; Gui et al. 2019; Cho et al. 2006; Feng et al. 2016; Zhang et al. 2018; Qiu et al. 2003; Zhu et al. 2012; Chi et al. 2014; Wang et al. 2015; Yamashiro et al. 2015; Cai et al. 2017; Li et al. 2017; Xu et al. 2017; Eroglu et al. 2018; Fu et al. 2019; Noiphithak et al. 2023; Xu et al. 2024; He et al. 2023; Lv et al. 2023; Wang et al. 2024; Kondabathini et al. 2024; Tahara et al. 2023; Yang et al. 2024; Fujita et al. 2021; Du et al. 2021; Zhang et al. 2014) including eight RCTs (Gui et al. 2019; Cho et al. 2006; Feng et al. 2016; Zhang et al. 2018; Noiphithak et al. 2023; Xu et al. 2024; Lv et al. 2023; Zhang et al. 2014) and 18 cohorts (Sun et al. 2019; Qiu et al. 2003; Zhu et al. 2012; Chi et al. 2014; Wang et al. 2015; Yamashiro et al. 2015; Cai et al. 2017; Li et al. 2017; Xu et al. 2017; Eroglu et al. 2018; Fu et al. 2019; He et al. 2023; Wang et al. 2024; Kondabathini et al. 2024; Tahara et al. 2023; Yang et al. 2024; Fujita et al. 2021; Du et al. 2021) comprising 1,251 events: 2,603 patients in the NE group (28%) and 6,562 patients in the CR group (72%) reported the mortality outcome. The NE approach significantly decreased the mortality with a pooled RR = 0.62 (95% CI: [0.48,0.81]; p < 0.0004; I^2^ = 51%). In the subgroup analysis, mortality was lower in the NE arm in the cohort's subgroup, but the results were insignificant in the RCT subgroup (**Figure** **3** **)**.

### 4.3 Hematoma Evacuation Rate

**FIGURE 4:**
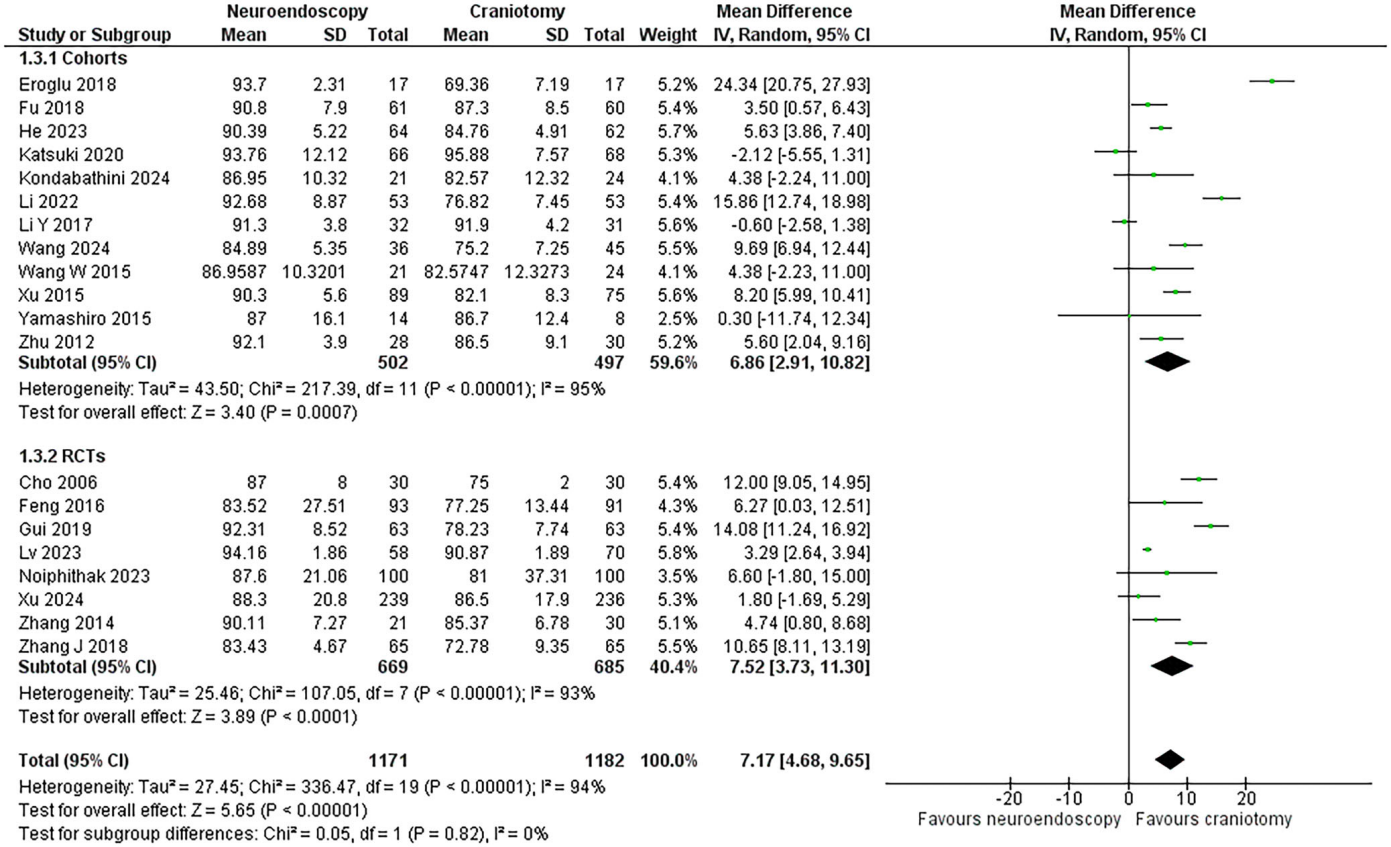
Hematoma evacuation rate forest plot.

This outcome was reported by 20 studies (Gui et al. 2019; Cho et al. 2006; Feng et al. 2016; Zhang et al. 2018; Zhu et al. 2012; Wang et al. 2015; Yamashiro et al. 2015; Li et al. 2017; Xu et al. 2017; Eroglu et al. 2018; Fu et al. 2019; Noiphithak et al. 2023; Xu et al. 2024; Li et al. 2022; He et al. 2023; Lv et al. 2023; Wang et al. 2024; Kondabathini et al. 2024; Katsuki et al. 2020; Zhang et al. 2014) with eight RCTs (Gui et al. 2019; Cho et al. 2006; Feng et al. 2016; Zhang et al. 2018; Noiphithak et al. 2023; Xu et al. 2024; Lv et al. 2023; Zhang et al. 2014) and 12 cohorts (Zhu et al. 2012; Wang et al. 2015; Yamashiro et al. 2015; Li et al. 2017; Xu et al. 2017; Eroglu et al. 2018; Fu et al. 2019; Li et al. 2022; He et al. 2023; Wang et al. 2024; Kondabathini et al. 2024; Katsuki et al. 2020) comprising 2,353 patients (1,171 NE versus 1,182 CR). The NE group was associated with an increased rate of hematoma evacuation compared to the CR (WMD = 7.17, 95% CI: [4.68, 9.65]; p < 0.00001). The heterogeneity was high (I^2^ = 94%; p < 0.00001). The results of the subgroup analysis remained similar (**Figure** **4**).

### 4.4 Operating Time

**FIGURE 5:**
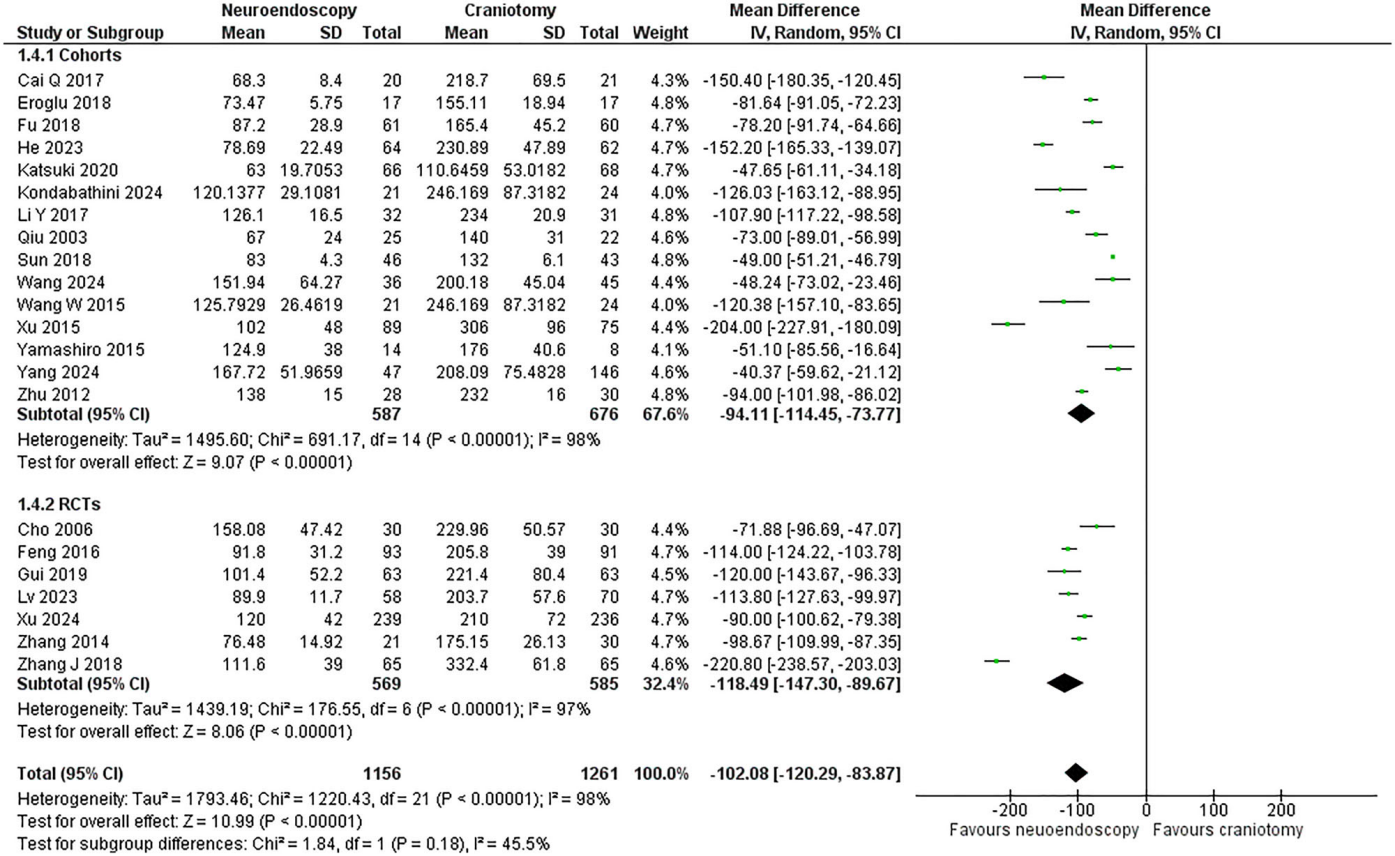
Operating Time Forest Plot.

Operating time was reported in 22 studies (Sun et al. 2019; Gui et al. 2019; Cho et al. 2006; Feng et al. 2016; Zhang et al. 2018; Qiu et al. 2003; Zhu et al. 2012; Wang et al. 2015; Yamashiro et al. 2015; Cai et al. 2017; Li et al. 2017; Xu et al. 2017; Eroglu et al. 2018; Fu et al. 2019; Xu et al. 2024; He et al. 2023; Lv et al. 2023; Wang et al. 2024; Kondabathini et al. 2024; Katsuki et al. 2020; Yang et al. 2024; Zhang et al. 2014)with seven RCTs (Gui et al. 2019; Cho et al. 2006; Feng et al. 2016; Zhang et al. 2018; Xu et al. 2024; Lv et al. 2023; Zhang et al. 2014)and 15 cohorts (Sun et al. 2019; Qiu et al. 2003; Zhu et al. 2012; Wang et al. 2015; Yamashiro et al. 2015; Cai et al. 2017; Li et al. 2017; Xu et al. 2017; Eroglu et al. 2018; Fu et al. 2019; He et al. 2023; Wang et al. 2024; Kondabathini et al. 2024; Katsuki et al. 2020; Yang et al. 2024) comprising 2,417 patients (1,156 NE versus 1,261 CR). The NE group was associated with a significantly decreased operating time (WMD = ‐102.08 min, 95 CI: [‐120.29, ‐83.87]; p < 0.00001). The heterogeneity was high (I^2^ = 98%; p < 0.00001). In the subgroup analysis, the NE arm remained superior regarding this outcome (**Figure** **5**).

### 4.5 Blood Loss

**FIGURE 6:**
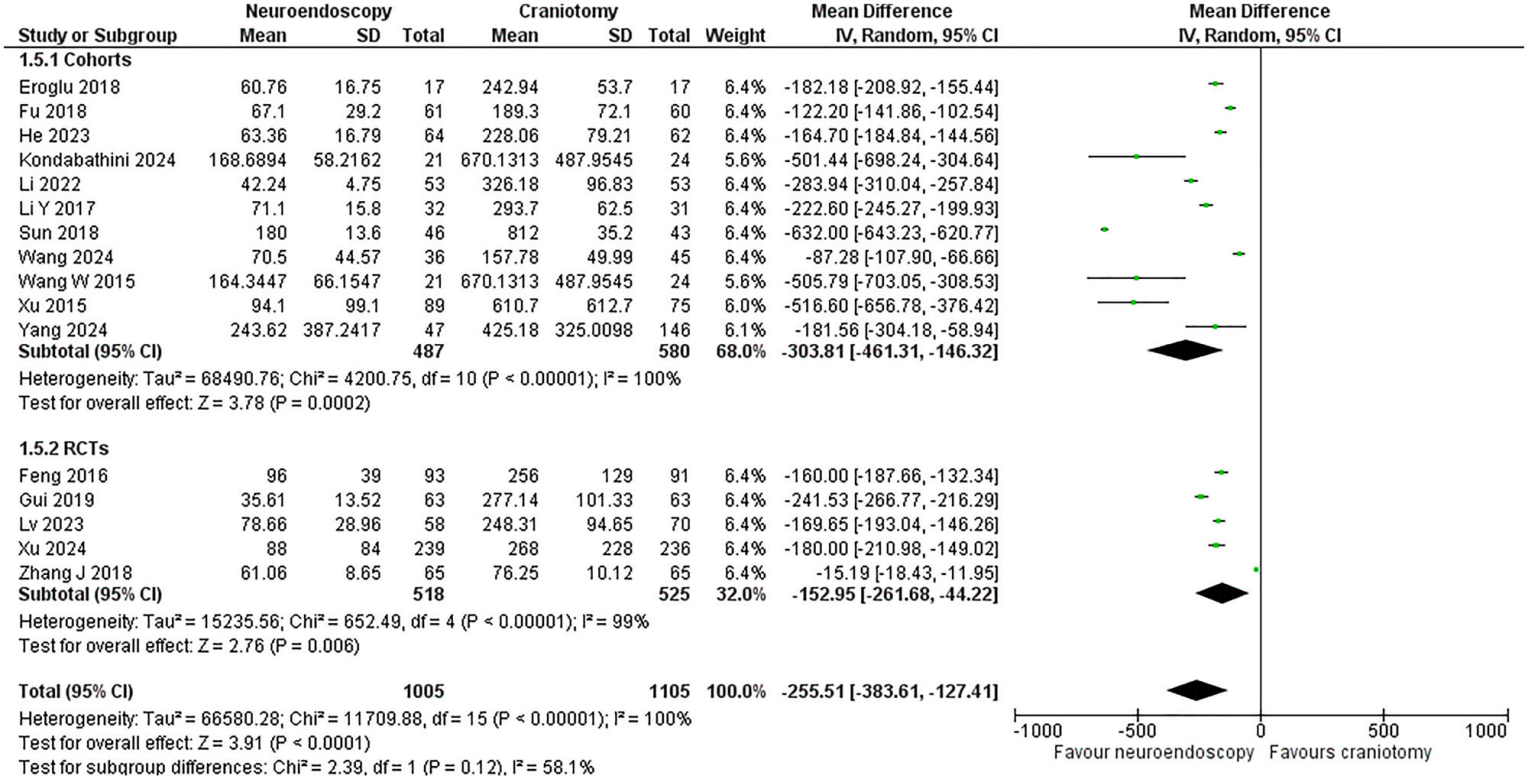
Blood Loss Forest Plot.

Sixteen studies (Sun et al. 2019; Gui et al. 2019; Feng et al. 2016; Zhang et al. 2018; Wang et al. 2015; Li et al. 2017; Xu et al. 2017; Eroglu et al. 2018; Fu et al. 2019; Xu et al. 2024; Li et al. 2022; He et al. 2023; Lv et al. 2023; Wang et al. 2024; Kondabathini et al. 2024; Yang et al. 2024) with five RCTs (Gui et al. 2019; Feng et al. 2016; Zhang et al. 2018; Xu et al. 2024; Lv et al. 2023)and 11 cohorts (Sun et al. 2019; Wang et al. 2015; Li et al. 2017; Xu et al. 2017; Eroglu et al. 2018; Fu et al. 2019; Li et al. 2022; He et al. 2023; Wang et al. 2024; Kondabathini et al. 2024; Yang et al. 2024) comprising 2,110 patients (1,005 NE versus 1,105 CR) reported this outcome. The NE group was significantly superior to the CR group in terms of this outcome (WMD = ‐255.51 mL, 95% CI: [‐383.61, ‐ 127.41]; p < 0.0001). The heterogeneity was very high (I^2^ = 100%; p < 0.00001). The NE arm maintained its superiority in subgrouping, as evidenced by a significant decrease in blood loss (**Figure** **6**).

### 4.6 Length of Hospitalization

**FIGURE 7:**
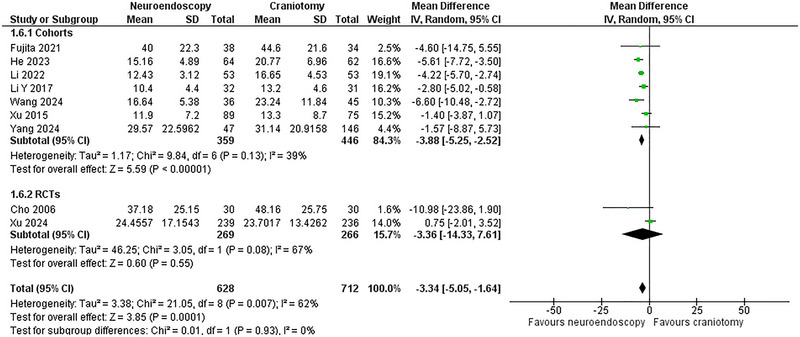
Length of Hospitalization Forest Plot.

Nine studies (Cho et al. 2006; Li et al. 2017; Xu et al. 2017; Xu et al. 2024; Li et al. 2022; He et al. 2023; Wang et al. 2024; Yang et al. 2024; Fujita et al. 2021) with two RCTs (Cho et al. 2006; Xu et al. 2024) and seven cohorts (Li et al. 2017; Xu et al. 2017; Li et al. 2022; He et al. 2023; Wang et al. 2024; Yang et al. 2024; Fujita et al. 2021), comprising 1,340 patients (628 NE versus 712 CR), reported this outcome. The NE group experienced a significantly shorter hospitalization length compared to the CR group (WMD = ‐3.34 days, 95% CI: [‐5.05,‐ 1.64]; p < 0.0001; I^2^ = 62%). In the subgroup analysis, the NE group maintained its superiority in the cohort subgroup, whereas the results were nonsignificant in the RCT subgroup (**Figure** **7**).

### 4.7 ICU Stay

Seven studies (Cho et al. 2006; Wang et al. 2015; Li et al. 2017; Eroglu et al. 2018; Xu et al. 2024; Lv et al. 2023; Yang et al. 2024) with three RCTs (Cho et al. 2006; Xu et al. 2024; Lv et al. 2023) and four cohorts (Wang et al. 2015; Li et al. 2017; Eroglu et al. 2018; Yang et al. 2024) comprising 998 patients (444 NE versus 554 CR) reported this outcome. The NE group significantly decreased the length of ICU stay (WMD = ‐2.85 days, 95% CI: [‐5.13, ‐0.57]; p < 0.01). The heterogeneity was very high (I^2^ = 96%; p < 0.00001). Regarding subgrouping, the NE group showed superiority in the cohort subgroup, whereas the results became non‐significant in the RCT subgroup (Supplementary Figure S2).

### 4.8 Rebleeding

A total of 18 studies (Sun et al. 2019; Cho et al. 2006; Zhu et al. 2012; Wang et al. 2015; Cai et al. 2017; Li et al. 2017; Eroglu et al. 2018; Fu et al. 2019; Noiphithak et al. 2023; Xu et al. 2024; Li et al. 2022; He et al. 2023; Wang et al. 2024; Kondabathini et al. 2024; Katsuki et al. 2020; Yang et al. 2024; Fujita et al. 2021; Zhang et al. 2014) with four RCTs (Cho et al. 2006; Noiphithak et al. 2023; Xu et al. 2024; Zhang et al. 2014) and 14 cohorts (Sun et al. 2019; Zhu et al. 2012; Wang et al. 2015; Cai et al. 2017; Li et al. 2017; Eroglu et al. 2018; Fu et al. 2019; Li et al. 2022; He et al. 2023; Wang et al. 2024; Kondabathini et al. 2024; Katsuki et al. 2020; Yang et al. 2024; Fujita et al. 2021) comprising 106 events: 940 patients in the NE group (47%) and 1,054 patients in the CR group (53%) reported the rebleeding outcome. Both groups were comparable regarding this outcome (RR = 0.76, 95% CI: [0.51, 1.12], p = 0.16; I^2 =^ 0%). On subgrouping, the results remained similar (Supplementary Figure S3).

### 4.9 Overall Complications

22 studies (Sun et al. 2019; Gui et al. 2019; Cho et al. 2006; Feng et al. 2016; Zhu et al. 2012; Wang et al. 2015; Cai et al. 2017; Li et al. 2017; Eroglu et al. 2018; Fu et al. 2019; Noiphithak et al. 2023; Xu et al. 2024; Li et al. 2022; He et al. 2023; Lv et al. 2023; Wang et al. 2024; Kondabathini et al. 2024; Katsuki et al. 2020; Tahara et al. 2023; Yang et al. 2024; Fujita et al. 2021; Zhang et al. 2014) with seven RCTs (Gui et al. 2019; Cho et al. 2006; Feng et al. 2016; Noiphithak et al. 2023; Xu et al. 2024; Lv et al. 2023; Zhang et al. 2014) and 15 cohorts (Sun et al. 2019; Zhu et al. 2012; Wang et al. 2015; Cai et al. 2017; Li et al. 2017; Eroglu et al. 2018; Fu et al. 2019; Li et al. 2022; He et al. 2023; Wang et al. 2024; Kondabathini et al. 2024; Katsuki et al. 2020; Tahara et al. 2023; Yang et al. 2024; Fujita et al. 2021) comprising 1,005 events: 2,049 patients in the NE group (26%) and 5,779 patients in the CR group (74%) reported this outcome. Overall complications were significantly lower in the NE group (RR = 0.52, 95% CI: [0.40,0.67]; p < 0.00001). The heterogeneity was high (I^2^ = 70 %; p < 0.00001). In the subgroup analysis, the results remained similar (Supplementary Figure S4).

### 4.10 Meningitis

Twelve studies (Gui et al. 2019; Cho et al. 2006; Li et al. 2017; Fu et al. 2019; Noiphithak et al. 2023; Xu et al. 2024; Li et al. 2022; He et al. 2023; Lv et al. 2023; Wang et al. 2024; Tahara et al. 2023; Yang et al. 2024) with five RCTs (Gui et al. 2019; Cho et al. 2006; Noiphithak et al. 2023; Xu et al. 2024; Lv et al. 2023)and seven cohorts (Li et al. 2017; Fu et al. 2019; Li et al. 2022; He et al. 2023; Wang et al. 2024; Tahara et al. 2023; Yang et al. 2024) comprising 128 events: 1,678 patients in the NE group (24%) and 5,397 patients in the CR group (76%) reported the meningitis outcome. Meningitis was significantly decreased in the NE group (RR = 0.58, 95% CI: [0.36, 0.95]; p < 0.03; I^2^ = 10 %). The NE group retained its superiority in the cohort subgroup, whereas the results were non‐significant in the RCT subgroup (Supplementary Figure S5).

### 4.11 Infections

16 studies (Gui et al. 2019; Cho et al. 2006; Feng et al. 2016; Zhu et al. 2012; Cai et al. 2017; Li et al. 2017; Eroglu et al. 2018; Fu et al. 2019; Noiphithak et al. 2023; Xu et al. 2024; Li et al. 2022; He et al. 2023; Lv et al. 2023; Tahara et al. 2023; Yang et al. 2024; Zhang et al. 2014) with seven RCTs (Gui et al. 2019; Cho et al. 2006; Feng et al. 2016; Noiphithak et al. 2023; Xu et al. 2024; Lv et al. 2023; Zhang et al. 2014) and nine cohorts (Zhu et al. 2012; Cai et al. 2017; Li et al. 2017; Eroglu et al. 2018; Fu et al. 2019; Li et al. 2022; He et al. 2023; Tahara et al. 2023; Yang et al. 2024) comprising 479 events: 1,821 patients in the NE group (25%) and 5,541 patients in the CR group (75%) reported this outcome. The NE group experienced significantly fewer infections compared to the CR group (RR = 0.49, 95% CI: [0.35, 0.67]; p < 0.0001, I^2^ = 51%). On subgrouping, the results remained similar, with the NE group retaining its superiority (Supplementary Figure S6).

### 4.12 Residual Hematoma Volume

Six studies (Cho et al. 2006; Zhang et al. 2018; Qiu et al. 2003; Noiphithak et al. 2023; Li et al. 2022; Lv et al. 2023)with four RCTs (Cho et al. 2006; Zhang et al. 2018; Noiphithak et al. 2023; Lv et al. 2023)and two cohorts (Qiu et al. 2003; Li et al. 2022) comprising 671 patients (331 NE versus 340 CR) were included in the analysis for this outcome. The NE group significantly decreased the residual hematoma volume with a pooled MD of ‐2.22 (95% CI: [‐3.37, ‐1.07], p < 0.0002).The overall heterogeneity was high(I^2^ = 90%, P < 0.00001).In the subgroup analysis, the NE group remained superior in the RCT group, whereas the results were nonsignificant in the cohort's subgroup (Supplementary Figure S7).

### 4.13 Sensitivity Analysis

We performed the leave‐one‐out sensitivity analysis for pooled analyses with heterogeneity >50%. On removing the study by Du et al. the heterogeneity in the mortality outcome decreased from 51% to 22% (Supplementary Figure S8), whereas by removing the study by Xu et al. for the length of hospitalization, the heterogeneity decreased significantly (I2 = 36%) (Supplementary Figure S9). Similarly, by removing the study by Eroglu et al. in ICU stay outcome, Noiphithak et al. in infections and Lv et al. in residual hematoma volume, the overall heterogeneity decreased to 74%, 36%, and 24%, respectively (Supplementary Figure S10‐12).

### 4.14 Publication Bias

For outcomes with more than 10 studies pooled, we assessed publication bias visually using a funnel plot and statistically using Egger's regression. The funnel plot visualization for the favorable neurological outcome showed evident asymmetry, which was confirmed by Egger's regression test as statistically significant (Supplementary Figure S13). Similarly, the Eggers regression test further confirmed a high risk of publication bias for operating time (Supplementary Figure **S14**). Likewise, the bias observed in the funnel plot for overall complications was statistically significant, as confirmed by Egger's regression test (Supplementary Figure S15). A similar trend of statistically significant publication bias was observed in meningitis (Supplementary Figure S16) and infections (Supplementary Figure S17). The detailed description of p‐values and t‐values in Egger's regression test is depicted in Supplementary Table S3.

### 4.15 Meta‐Regression

Meta‐regression analysis was performed to assess the effect of covariates on the statistical outcome findings. Covariates utilized for the meta‐regression analysis include mean age, sample size, ICH volume, and preoperative GCS score. A statistically significant linear correlation was found between the mean ICH volume and hematoma evacuation rate for the CR group (Omnibus, p = 0.026) (Supplementary Figure S18). A significant correlation was also noted between the GCS and hematoma evacuation rate in the NE (Omnibus p = 0.001) and CR groups (Omnibus p = 0.000) (Supplementary Figure S19‐20). Similarly, a significant trend was also found between mean age and blood loss in the NE (Omnibus p = 0.012) and CR (Omnibus p = 0.04) groups (Supplementary Figure S21‐22).

## 5 Discussion

NE evacuation was initially suggested by Auer et al. and has since developed into a potentially effective surgical technique for managing spontaneous supratentorial ICH due to its high evacuation rate, superior protection of the surrounding brain tissue, and lower complication rates (Ye et al. 2017; Zhang et al. 2014). Both NE and CR have been proven to be successful surgical techniques for supratentorial ICH and are widely used. Numerous investigations have been conducted to evaluate the relative merits of CR and NE, yielding varying conclusions (Monteiro G. de et al. 2024; Sun et al. 2020; Guo et al. 2020; Zhao et al. 2019; Du et al. 2022; Hallenberger et al. 2022). By combining all these elements, we can provide a comprehensive view.

We conducted a systematic review and meta‐analysis to evaluate the safety and efficacy of NE compared to CR in the management of spontaneous supratentorial intracerebral hemorrhage (ICH). In response to the publication of new studies, we conducted the most up‐to‐date meta‐analysis on the subject, including the most recent cohorts and RCTs. After analyzing 28 studies, we found that NE performed better than traditional CR in several important domains. Firstly, NE demonstrated a notable advantage over CR in obtaining favorable neurological functional outcomes and reducing mortality. This result aligns with the findings of previous studies (Monteiro G. de et al. 2024; Sun et al. 2020; Guo et al. 2020; Du et al. 2022) and may be attributed to a lower incidence of complications, a higher rate of hematoma evacuation, shorter surgery durations, and fewer surgery‐related injuries in the NE group. However, Hallenberger et al. reported no significant difference between NE and CR in terms of favorable neurological function and death.

Another important measure of functional outcome is the rate of hematoma evacuation. According to our findings, there was a smaller residual hematoma volume in the NE group due to a significantly higher evacuation rate than in CR. Hallenberger et al. did not report any difference in the hematoma evacuation rate or residual hematoma volume, while the evacuation rate findings of Xu et al. supported our analysis. Compared to previous studies, we were able to find some new results. Rebleeding, meningitis, and infections with common overall complications were reported to be lower among patients who had NE than CR. Like our results, overall complications were consistently found to be lower in the NE group in previous studies; however, no significant findings have been established previously regarding rebleeding and infection rates (Monteiro G. de et al. 2024; Sun et al. 2020; Guo et al. 2020; Zhao et al. 2019; Du et al. 2022; Hallenberger et al. 2022). Compared to the CR group, NE was also linked to shorter operating times, lower blood loss, shorter hospital stays, and shorter stays in the intensive care unit.

NE has many advantages over CR. The multi‐angle observation, made possible by NE, compensates for the limitations of direct vision and aids in the management of intraoperative bleeding. (Feng et al. 2016). Traditional CRs are more traumatic and have the potential to permanently harm the brain's tissue as well as the blood vessels in the fistula (Du et al. 2022). Because NE avoids important brain functional regions, it more closely adheres to the principles of minimally invasive surgery (Lu et al. 2022). NE surgery is also able to locate and expose the hematoma without exposing unrelated tissues. (Zhang et al. 2014). NE uses internal lighting that allows for closer observation, whereas CR relies on exterior lighting that might not be bright enough for deep hematomas. (Zhou et al. 2024). Because the brightness doesn't change when the hematoma depth does, it makes the intraoperative conditions easier to see and improves procedural accuracy (Zhou et al. 2024). Additionally, a smaller incision, less brain tissue damage, and a shorter operation duration all lower the risk of infection (Zhang et al. 2014).

While NE has demonstrated significant benefits in the management of spontaneous supratentorial ICH, there is limited data comparing its efficacy in lobar versus deep supratentorial ICH. Existing studies suggest that NE offers advantages in terms of reduced surgical trauma, faster recovery times, and fewer complications, particularly in lobar ICH cases. These benefits are primarily attributed to the less invasive nature of NE, which facilitates better hematoma evacuation and improved functional outcomes in lobar regions. However, in deep ICH, such as basal ganglia hemorrhages, the technical challenges increase, and the outcomes are more variable. The deeper location and complex anatomy in these regions make successful NE evacuation more difficult, which may impact the overall results. Therefore, more targeted studies are needed to compare NE outcomes in lobar versus deep supratentorial ICH to better understand the differential efficacy of NE in these subtypes.

Previous studies faced several limitations that underscored the need for our research. Many analyses included a limited number of studies, often relying on non‐randomized controlled trials (non‐RCTs) that lacked random sequence generation or allocation concealment and excluded newly published studies, potentially increasing outcome bias. Additionally, the number of outcome indicators used for evaluation was often limited, weakening the validity of their conclusions. For example, some meta‐analyses failed to distinguish between supratentorial and infratentorial hemorrhages and combined different treatment methods (e.g., conservative management, craniotomy, aspiration, and neuroendoscopic surgery) in their subgroup analyses, further reducing the validity of the results. Another review even combined stereotactic drilling and NE surgery into a single category of minimally invasive surgery, despite their distinct processes and characteristics, leading to increased outcome bias.

To address these issues, our study included a broader range of studies, particularly by incorporating newly published research and prioritizing high‐quality RCTs. This approach enabled us to conduct meaningful subgroup and regression analyses, reduce bias, and provide a more comprehensive view. We also expanded the number of outcome indicators to enhance the robustness and validity of our conclusions. By focusing exclusively on supratentorial hemorrhages, we avoided conflating different conditions and carefully avoided combining different treatment methods in subgroup analyses, recognizing that these procedures have distinct characteristics and outcomes.

However, our study had some limitations that warrant consideration. Despite incorporating new RCTs into our analysis, several small sample‐size studies were included, which may affect the overall reliability of the findings. Because the primary outcome, a favorable neurological functional outcome, was reported at different times and using different measures, there was a risk of bias, which made direct comparisons more challenging. As a result, the results needed to be interpreted carefully. There was a lot of observed heterogeneity, most of which was likely due to variations between the studies, and it was addressed. Additionally, we limited the studies screened to those published in English, which may have introduced selection bias. The variations in follow‐up periods among studies make it difficult to draw consistent conclusions. Despite these challenges, our comprehensive approach has allowed us to present a more robust comparison of NE and CR, emphasizing the potential of NE as a preferable option for patients with supratentorial ICH. Future research with standardized follow‐up periods and larger patient cohorts will further strengthen the evidence base and refine treatment strategies.

## 6 Conclusion

Our systematic review and meta‐analysis provide moderate evidence supporting the use of NE as an effective surgical technique for managing spontaneous supratentorial ICH. NE showed several advantages over traditional CR, including better neurological outcomes, reduced mortality, higher rates of hematoma evacuation, and fewer complications and infections. These findings align with previous research and suggest that NE offers benefits such as improved protection of brain tissue, reduced invasiveness, and enhanced intraoperative visibility. More high‐quality, multi‐center RCTs are needed to strengthen the evidence and draw definitive conclusions.

## Author Contributions

**Muhammad Hassan Waseem**: Conceptualization; Writing ‐ original draft; Methodology; Writing ‐ review & editing. **Zain ul Abideen**: Investigation; Writing ‐ original draft; Formal analysis; Supervision. **Nohela Rehman**: Writing ‐ original draft; Writing ‐ review & editing. **Muhammad Haris Khan**: Methodology; Visualization; Software. **Muhammad Fawad Tahir**: Data curation; Resources; Software. **Hafsa Arshad Azam Raja**: Visualization; Resources; Data curation; Project administration. **Ameer Haider Cheema**: Writing ‐ review & editing; Funding acquisition; Supervision. **Sania Aimen**: Visualization; Software; Validation. **Javed Iqbal**: Writing ‐ review & editing; Project administration.

## Ethics Statement

The authors have nothing to report.

## Consent

The authors have nothing to report.

## Conflicts of Interest

The authors declare no conflicts of interest

## Peer Review

The peer review history for this article is available at https://publons.com/publon/10.1002/brb3.70581

## Supporting information



Supplementary Materials

## Data Availability

The corresponding author will make data available upon request, excluding confidential information.
