# Supplementary material for: Efficacy and Safety of Neuroendoscopy versus Craniotomy for Spontaneous Supratentorial Intracerebral Hemorrhage: An Updated Meta‐Analysis of Randomized and Non‐Randomized Studies
Source: Brain Behav. 2025 Sep 1;15(9):e70581. doi: 10.1002/brb3.70581 (PMC12400937; doi:10.1002/brb3.70581)
Supplement: Supplementary file 1 — Supplementary Materials [file BRB3-15-e70581-s001.docx]

**Title: Efficacy and Safety of Neuroendoscopy versus Craniotomy for Spontaneous Supratentorial Intracerebral Hemorrhage: An Updated Meta-analysis of Randomized and Non-randomized Studies**

**Running Head:** Neuroendoscopy versus Craniotomy for Intracerebral Hemorrhage

**Authors:**

Muhammad Hassan Waseem^1^, Zain Ul Abideen^2^, Nohela Rehman^3^, Muhammad Haris Khan^4^, Muhammad Fawad Tahir^5^, Hafsa Arshad Azam Raja^6^, Sania Aimen^7^

**Affiliations:**

**^1^** Allama Iqbal Medical College, Lahore, Pakistan

**^2^** King Edward Medical University, Lahore, Pakistan

**^3^** Dow University of Health Sciences, Karachi, Pakistan

**^4^** Saidu Medical College, Swat, Pakistan

**^5^** H.B.S Medical and Dental College, Islamabad, Pakistan

**^6^** Rawalpindi Medical University, Pakistan

^7^ Quetta Institute of Medical Sciences, Quetta, Pakistan

**Corresponding Author**

Zain ul Abideen

[mzainulabideen@kemu.edu.pk](mailto:mzainulabideen@kemu.edu.pk)

ORCID ID: 0009-0001-8990-7738

King Edward Medical University, Lahore, Pakistan  H897+X5V Chowk, Nila Gumbad Rd, Neela Gumbad Lahore, Punjab 54000

| **Supplementary Table S1:** Detailed search strategies used on different electronic databases | | |
| --- | --- | --- |
| **Database used** | **Search string** | **Articles retrieved** |
| PubMed | ("Neuroendoscopy"[MeSH Terms] OR ("Neuroendoscopy"[MeSH Terms] OR "Neuroendoscopy"[All Fields] OR "neuroendoscopies"[All Fields])) AND ("Intracranial Hemorrhages"[MeSH Terms] OR ("Intracranial Hemorrhages"[MeSH Terms] OR ("intracranial"[All Fields] AND "hemorrhages"[All Fields]) OR "Intracranial Hemorrhages"[All Fields] OR ("hemorrhages"[All Fields] AND "intracranial"[All Fields]) OR "hemorrhages intracranial"[All Fields]) OR ("intracranial haemorrhage"[All Fields] OR "Intracranial Hemorrhages"[MeSH Terms] OR ("intracranial"[All Fields] AND "hemorrhages"[All Fields]) OR "Intracranial Hemorrhages"[All Fields] OR ("intracranial"[All Fields] AND "hemorrhage"[All Fields]) OR "intracranial hemorrhage"[All Fields]) OR ("Intracranial Hemorrhages"[MeSH Terms] OR ("intracranial"[All Fields] AND "hemorrhages"[All Fields]) OR "Intracranial Hemorrhages"[All Fields] OR ("hemorrhage"[All Fields] AND "intracranial"[All Fields]) OR "hemorrhage intracranial"[All Fields]) OR ("brain haemorrhage"[All Fields] OR "Intracranial Hemorrhages"[MeSH Terms] OR ("intracranial"[All Fields] AND "hemorrhages"[All Fields]) OR "Intracranial Hemorrhages"[All Fields] OR ("brain"[All Fields] AND "hemorrhage"[All Fields]) OR "brain hemorrhage"[All Fields]) OR ("brain haemorrhages"[All Fields] OR "Intracranial Hemorrhages"[MeSH Terms] OR ("intracranial"[All Fields] AND "hemorrhages"[All Fields]) OR "Intracranial Hemorrhages"[All Fields] OR ("brain"[All Fields] AND "hemorrhages"[All Fields]) OR "brain hemorrhages"[All Fields]) OR ("Intracranial Hemorrhages"[MeSH Terms] OR ("intracranial"[All Fields] AND "hemorrhages"[All Fields]) OR "Intracranial Hemorrhages"[All Fields] OR ("hemorrhage"[All Fields] AND "brain"[All Fields]) OR "hemorrhage brain"[All Fields]) OR ("Intracranial Hemorrhages"[MeSH Terms] OR ("intracranial"[All Fields] AND "hemorrhages"[All Fields]) OR "Intracranial Hemorrhages"[All Fields] OR ("hemorrhages"[All Fields] AND "brain"[All Fields]) OR "hemorrhages brain"[All Fields]) OR ("posterior fossa haemorrhage"[All Fields] OR "Intracranial Hemorrhages"[MeSH Terms] OR ("intracranial"[All Fields] AND "hemorrhages"[All Fields]) OR "Intracranial Hemorrhages"[All Fields] OR ("posterior"[All Fields] AND "fossa"[All Fields] AND "hemorrhage"[All Fields]) OR "posterior fossa hemorrhage"[All Fields]) OR ("Intracranial Hemorrhages"[MeSH Terms] OR ("intracranial"[All Fields] AND "hemorrhages"[All Fields]) OR "Intracranial Hemorrhages"[All Fields] OR ("hemorrhage"[All Fields] AND "posterior"[All Fields] AND "fossa"[All Fields]) OR "hemorrhage posterior fossa"[All Fields]) OR ("Intracranial Hemorrhages"[MeSH Terms] OR ("intracranial"[All Fields] AND "hemorrhages"[All Fields]) OR "Intracranial Hemorrhages"[All Fields] OR ("hemorrhages"[All Fields] AND "posterior"[All Fields] AND "fossa"[All Fields])) OR ("posterior fossa haemorrhages"[All Fields] OR "Intracranial Hemorrhages"[MeSH Terms] OR ("intracranial"[All Fields] AND "hemorrhages"[All Fields]) OR "Intracranial Hemorrhages"[All Fields] OR ("posterior"[All Fields] AND "fossa"[All Fields] AND "hemorrhages"[All Fields]) OR "posterior fossa hemorrhages"[All Fields])) | 398 |
| Cochrane Central | ("Neuroendoscopy"[Mesh] OR Neuroendoscopies) AND ("Intracranial Hemorrhages"[Mesh] OR Hemorrhages, Intracranial OR Intracranial Hemorrhage OR Hemorrhage, Intracranial OR Brain Hemorrhage OR Brain Hemorrhages OR Hemorrhage, Brain OR Hemorrhages, Brain OR Posterior Fossa Hemorrhage OR Hemorrhage, Posterior Fossa OR Hemorrhages, Posterior Fossa OR Posterior Fossa Hemorrhages) | 31 |
| ScienceDirect | (Neuroendoscopy) AND (Intracranial Hemorrhage OR Hemorrhages, Intracranial OR Intracranial Hemorrhage OR Hemorrhage, Intracranial OR Brain Hemorrhage OR Brain Hemorrhages OR Hemorrhage, Brain OR Hemorrhages, Brain) | 497 |

| **Supplementary Table S2:** Newcastle Ottawa Scale for the quality assessment of the non-randomized studies | | | | | | | | | | |
| --- | --- | --- | --- | --- | --- | --- | --- | --- | --- | --- |
| **Cohort Studies** | | **Selection** | | | | **Comparability** | **Exposure** | | | **Total** |
| ***Author*** | ***Year*** | ***Representativeness of Exposed Cohort*** | ***Selection of the Non exposed cohort*** | ***Ascertainment of Exposure*** | ***Outcome was not Present at the start of Study*** |  | ***Assessment of Outcome*** | ***Follow-up long enough for Outcomes to occur*** | ***Adequacy of follow-up of Cohorts*** |  |
| Qui | 2003 | * | * | * |  | ** | * | * |  | 7 |
| Zhu | 2011 | * | * | * | * | ** | * | * |  | 8 |
| Chi | 2014 | * | * | * |  | ** | * | * | * | 8 |
| Wang W | 2015 | * | * | * |  | * | * | * | * | 7 |
| Yamashiro | 2015 | * | * | * | * | * | * | * |  | 7 |
| Cai Q | 2017 | * | * | * | * | * | * | * | * | 8 |
| Li Y | 2017 | * | * | * |  | * | * |  | * | 6 |
| Xu | 2015 | * | * | * | * | * | * | * | * | 8 |
| Eroglu | 2018 | * | * | * | * | * | * | * | * | 8 |
| Fu | 2018 | * | * | * | * | * | * | * | * | 8 |
| Sun | 2018 | * | * | * | * | * | * | * |  | 7 |
| Li | 2022 | * | * | * | * | * | * | * | * | 8 |
| He | 2023 | * | * | * |  | * * | * | * |  | 7 |
| Wang | 2024 | * | * | * | * | * | * | * | * | 8 |
| Kondabathini | 2024 | * | * | * | * | * | * | * |  | 7 |
| Katsuki | 2020 | * | * | * |  | ** | * | * |  | 7 |
| Tahara | 2023 | * | * | * |  | * | * | * | * | 7 |
| Yang | 2024 | * | * | * |  | * | * | * | * | 7 |
| Du | 2021 | * | * | * |  | * | * | * | * | 7 |
| Fujita | 2021 | * | * | * |  | * | * | * | * | 7 |

| **Supplementary Table S3:** Egger regression test | | |
| --- | --- | --- |
| **Outcome** | **P-value** | **t-value** |
| Favourable Neurological Outcome | 0.00670 | 3.11257 |
| Operating time | 0.00046 | 4.18120 |
| Overall Complications | 0.04452 | 2.15986 |
| Meningitis | 0.00598 | 3.47371 |
| Infections | 0.02008 | 2.62245 |


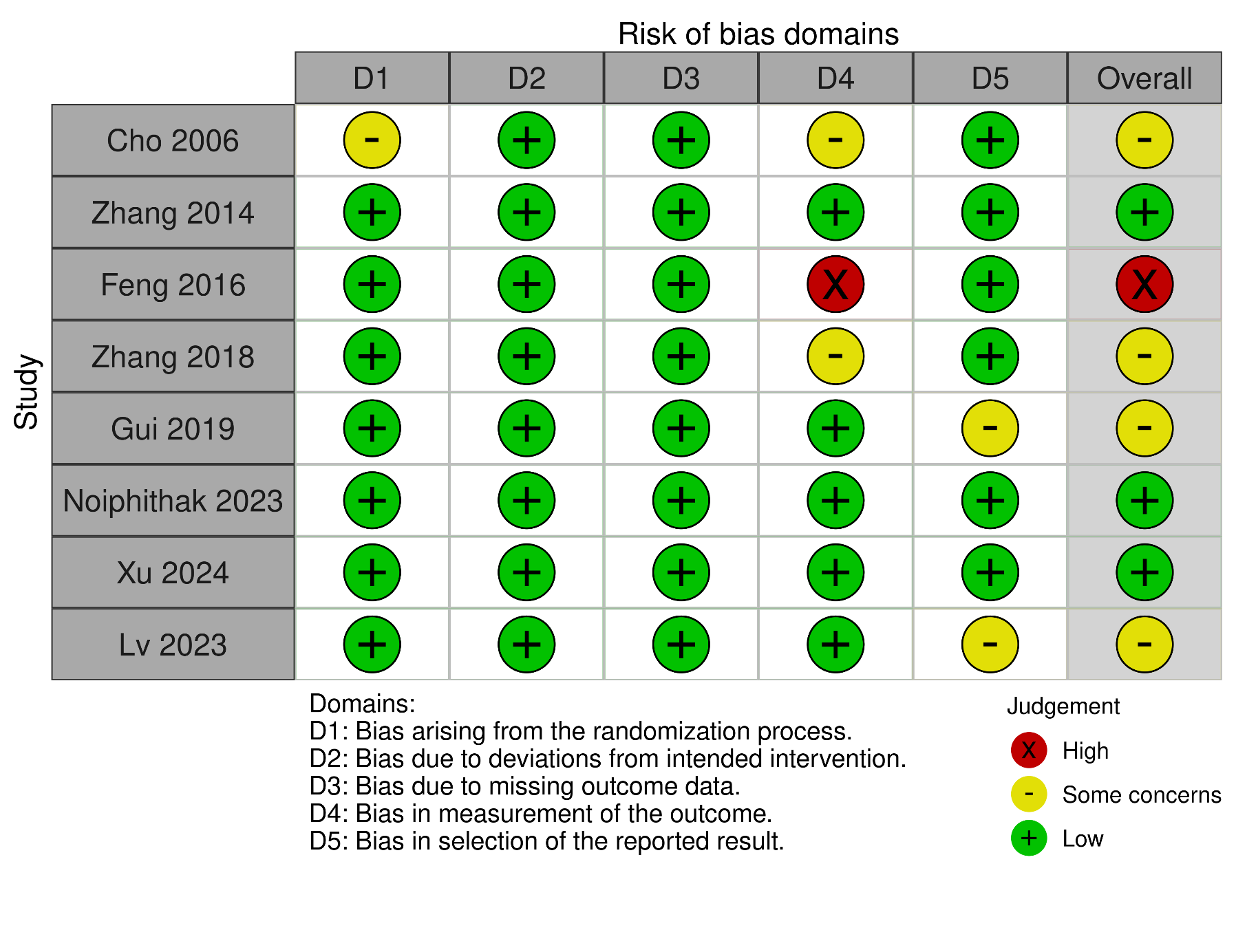
**Supplementary Figure S1** RoB 2 Quality assessment Traffic plot

**
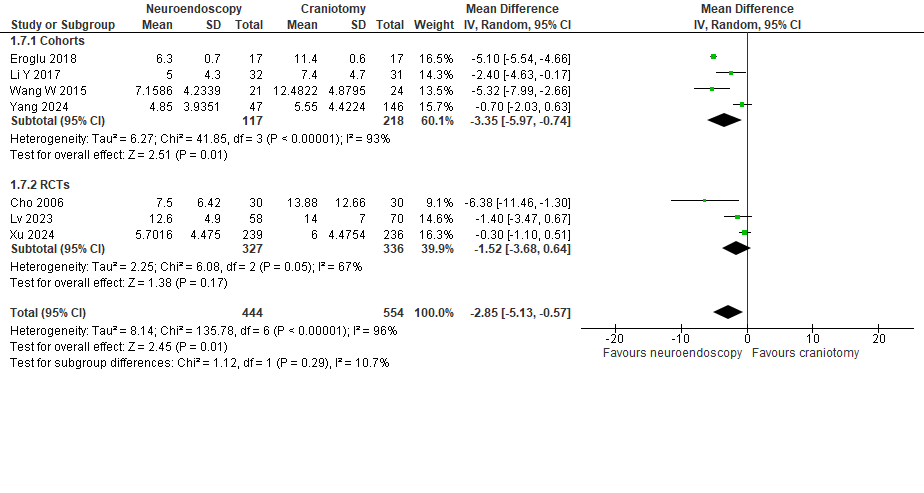
Supplementary Figure S2** ICU stay Forest Plot

**
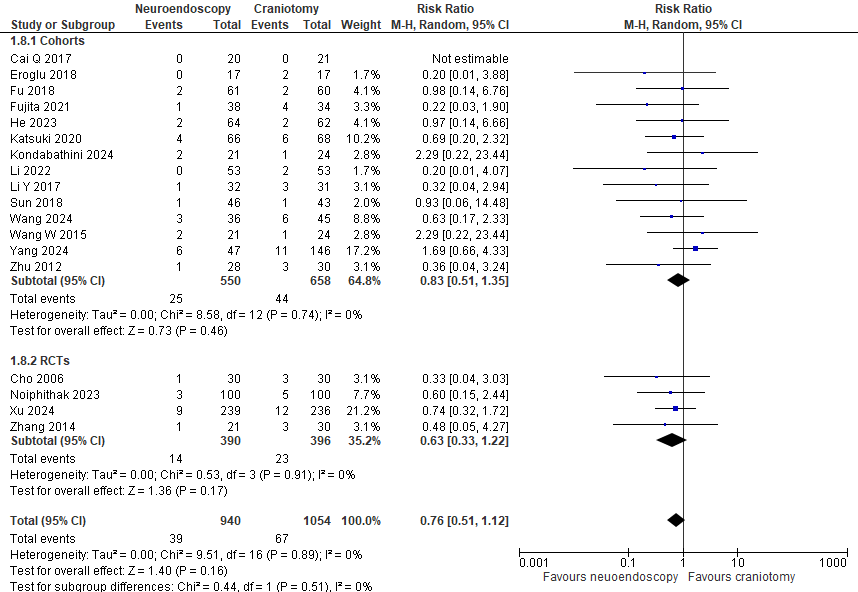
**

**Supplementary Figure S3** Rebleeding Forest Plot

**
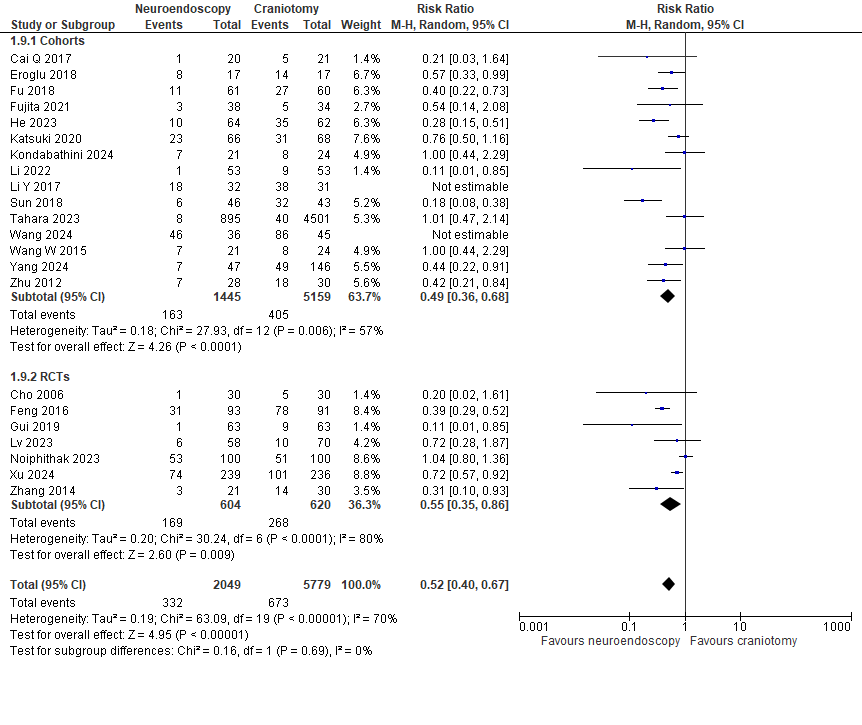
Supplementary Figure S4** Overall Complications Forest Plot

**
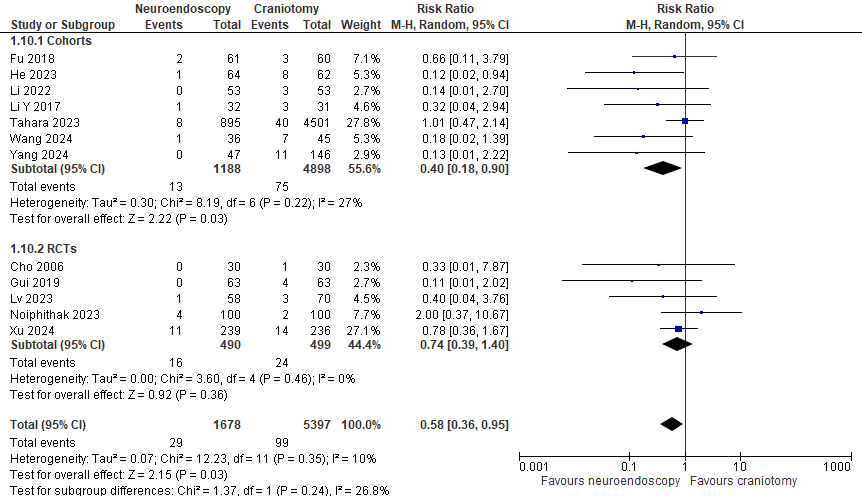
**

**Supplementary Figure S5** Meningitis Forest Plot

**
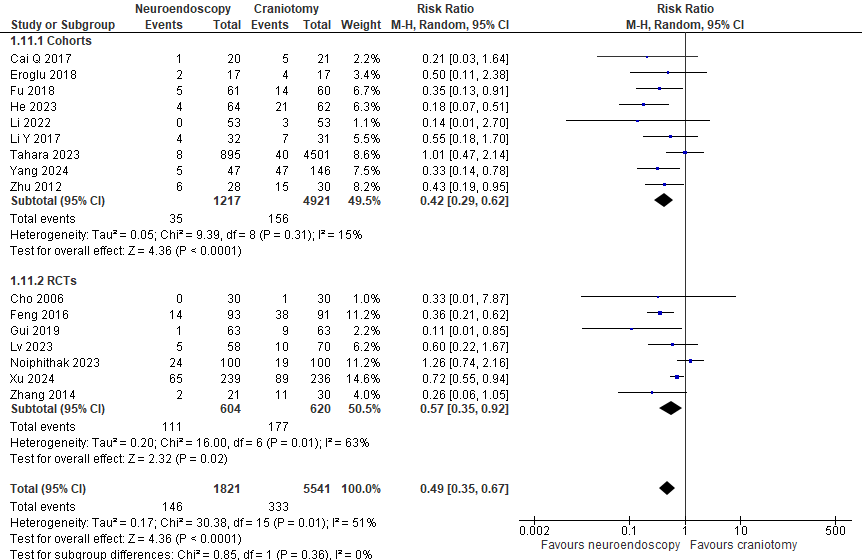
**

**Supplementary Figure S6** Infections Forest Plot

**
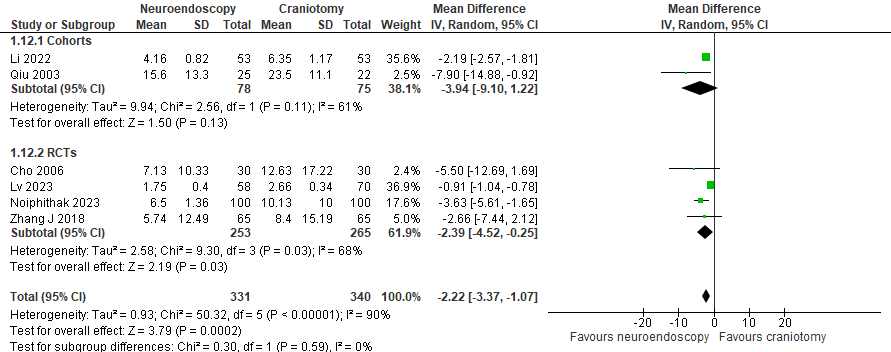
**

**Supplementary Figure S7** Residual Hematoma Volume Forest Plot


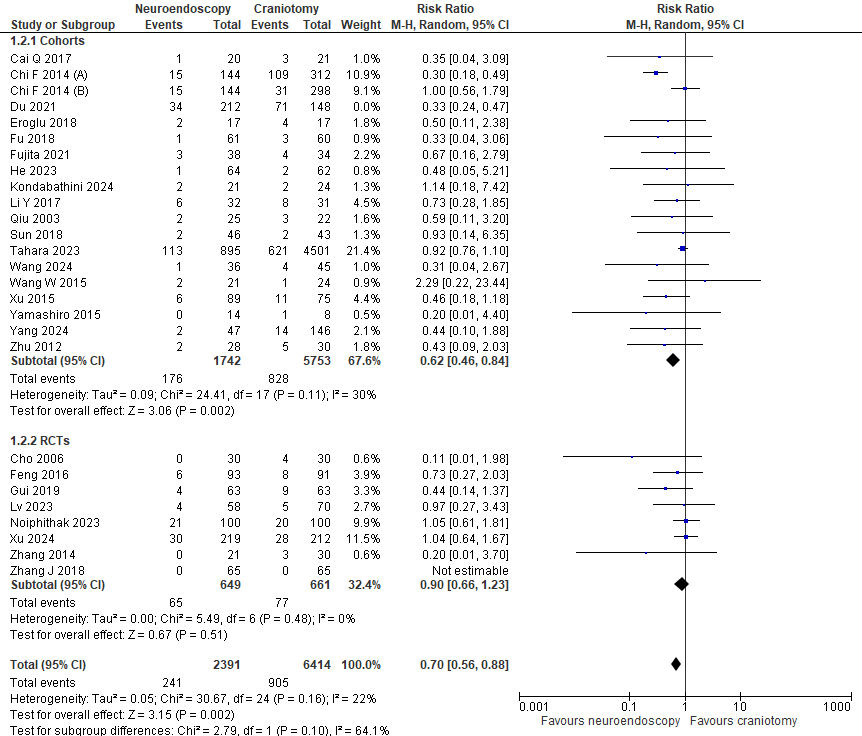


**Supplementary Figure S8** Mortality Leave-one-out Sensitivity Forest plot


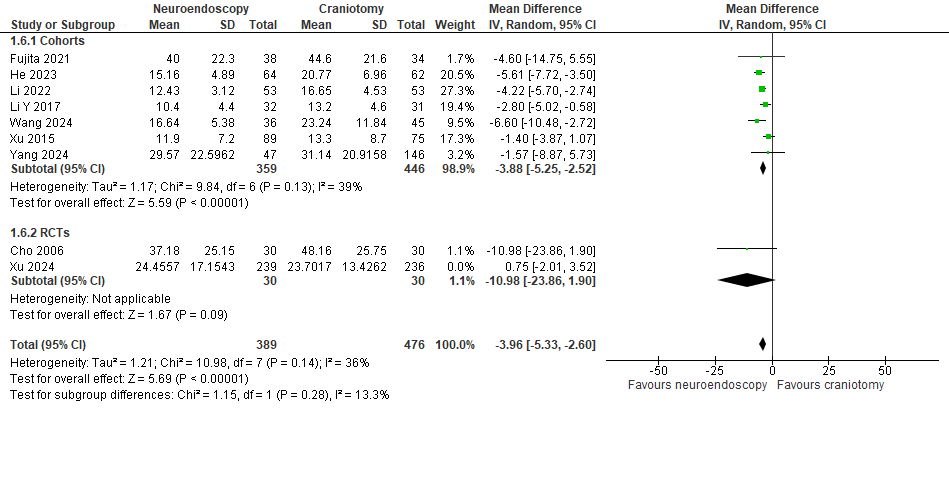
**Supplementary Figure S9** Length of Hospitalization Leave-one-out Sensitivity Forest plot


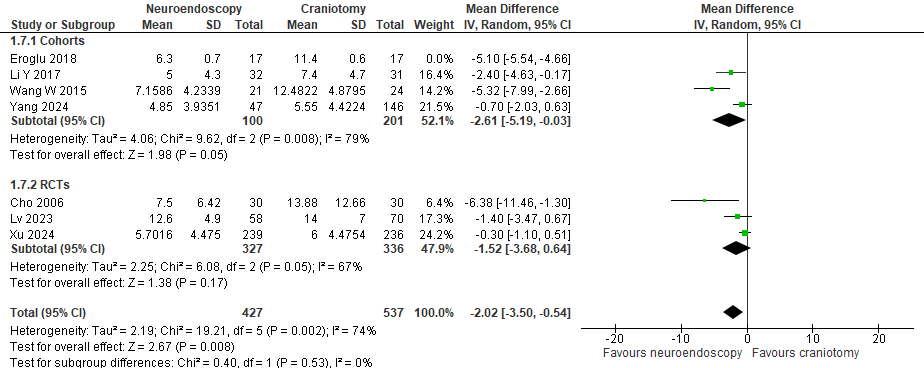


**Supplementary Figure S10** ICU stay Leave-one-out Sensitivity Forest plot


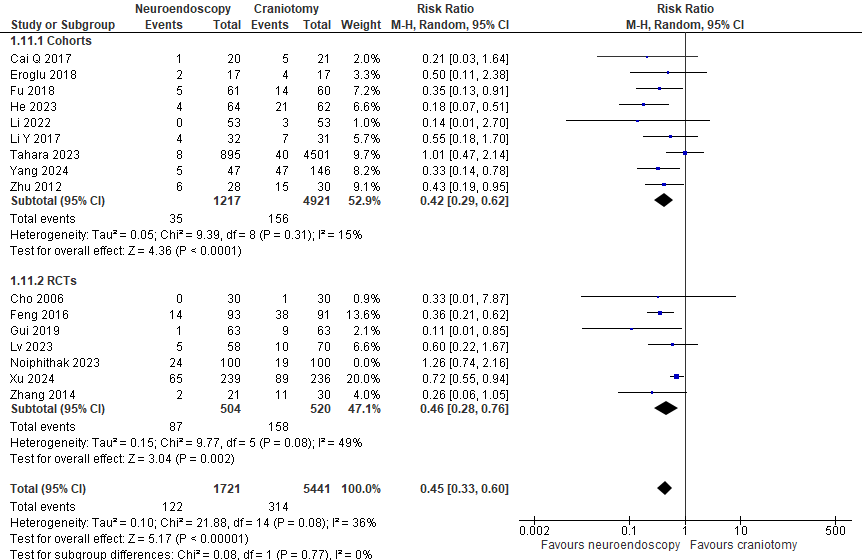


**Supplementary Figure S11** Infections Leave-one-out Sensitivity Forest plot


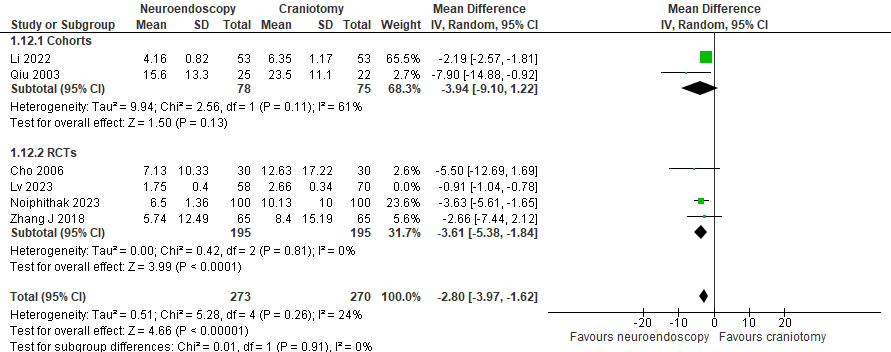


**Supplementary Figure S12** Residual Hematoma Volume Leave-one-out Sensitivity Forest plot


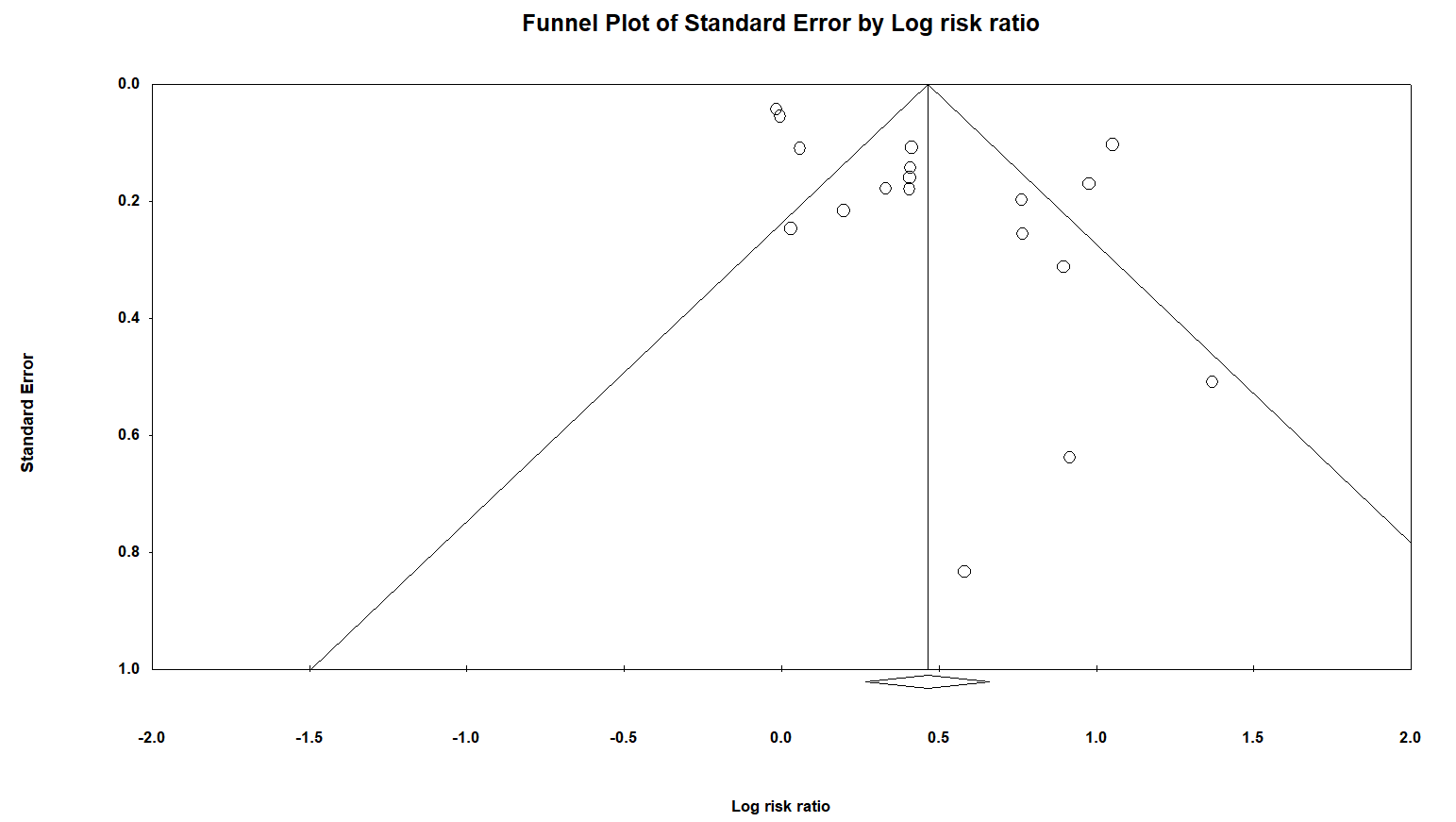


**Supplementary Figure S13** Favourable Neurological Outcome Funnel plot


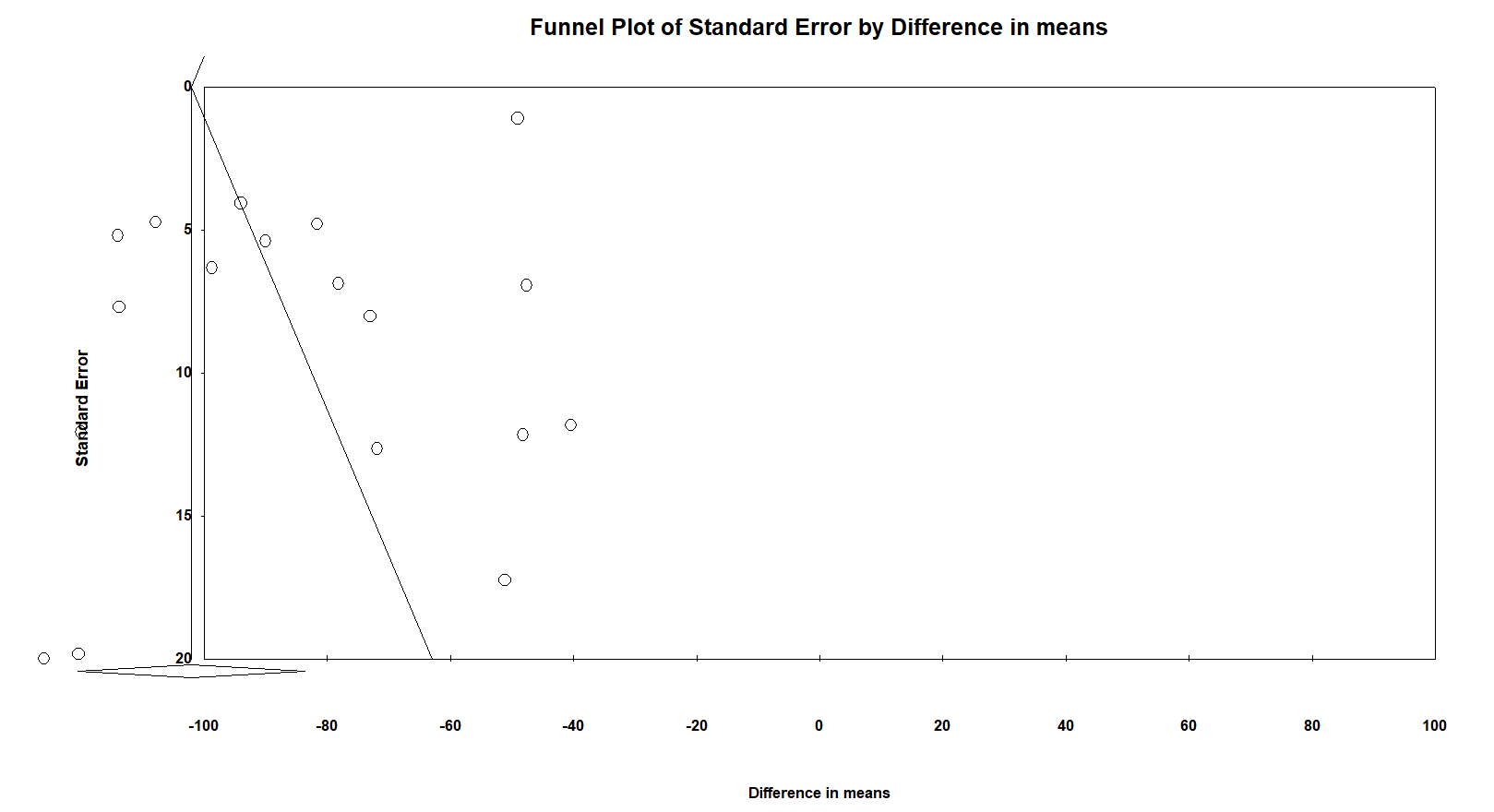


**Supplementary Figure S14** Operating time Funnel Plot


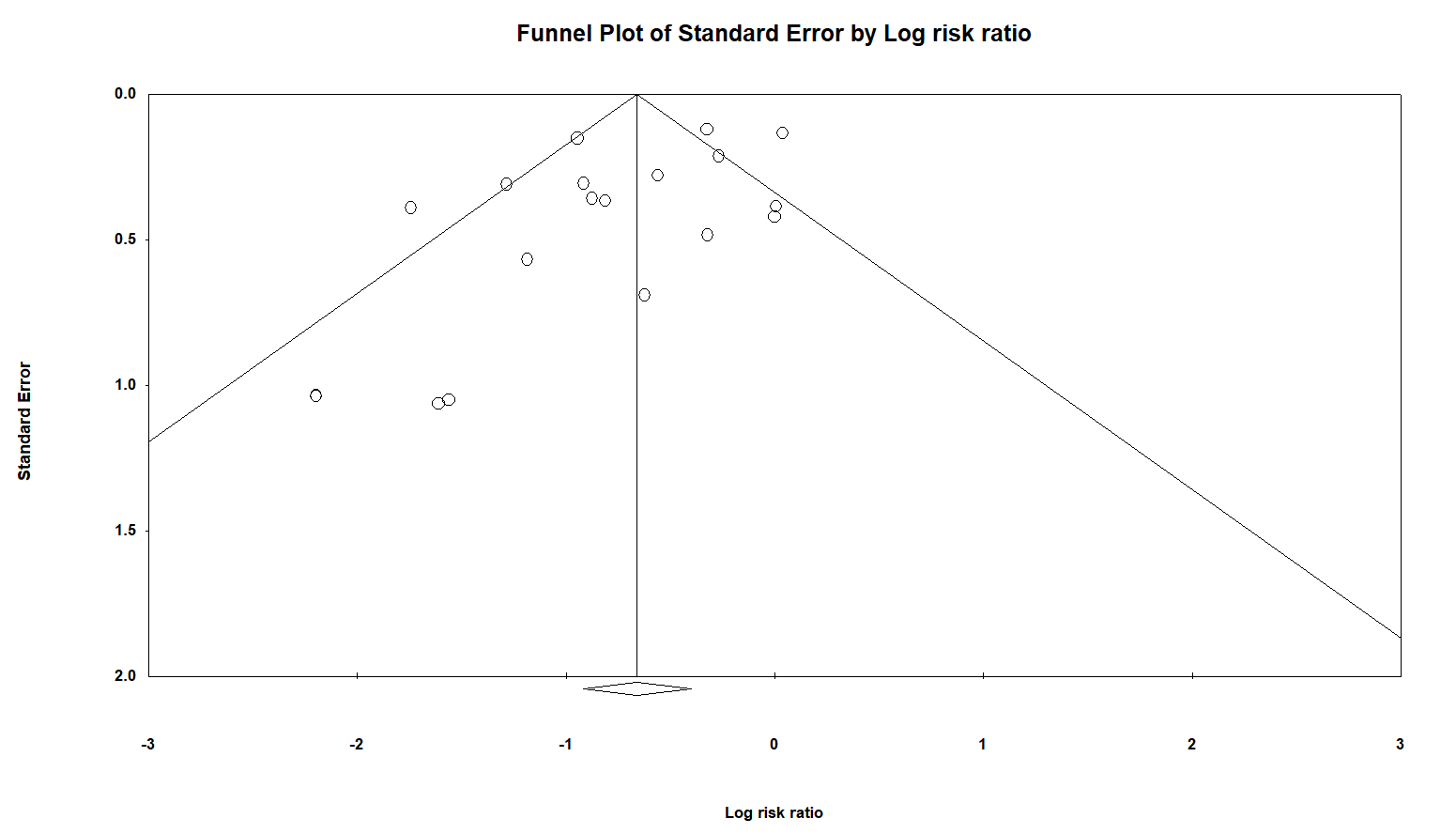


**Supplementary Figure S15** Overall Complications Funnel Plot


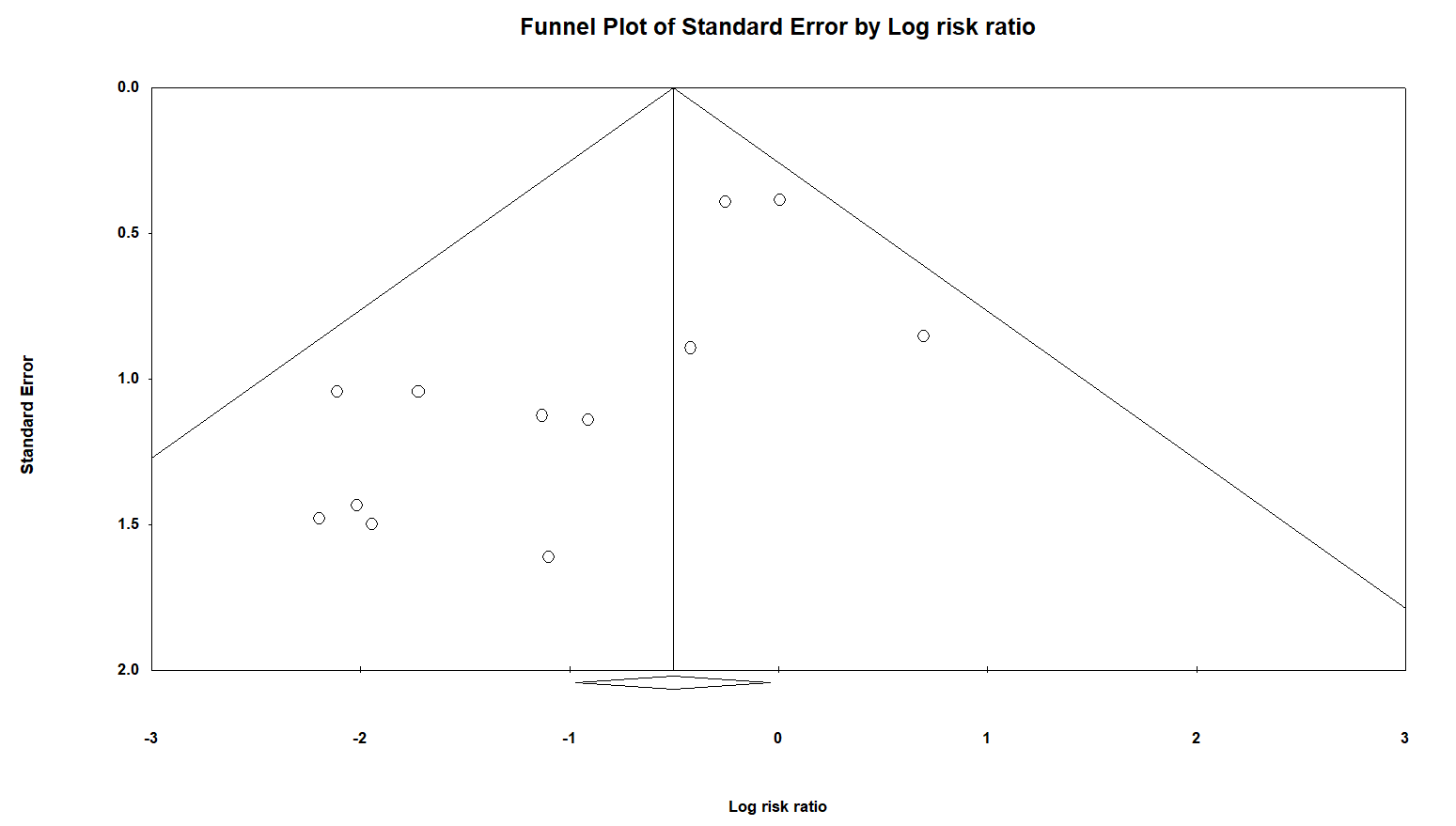


**Supplementary Figure S16** Meningitis Funnel Plot


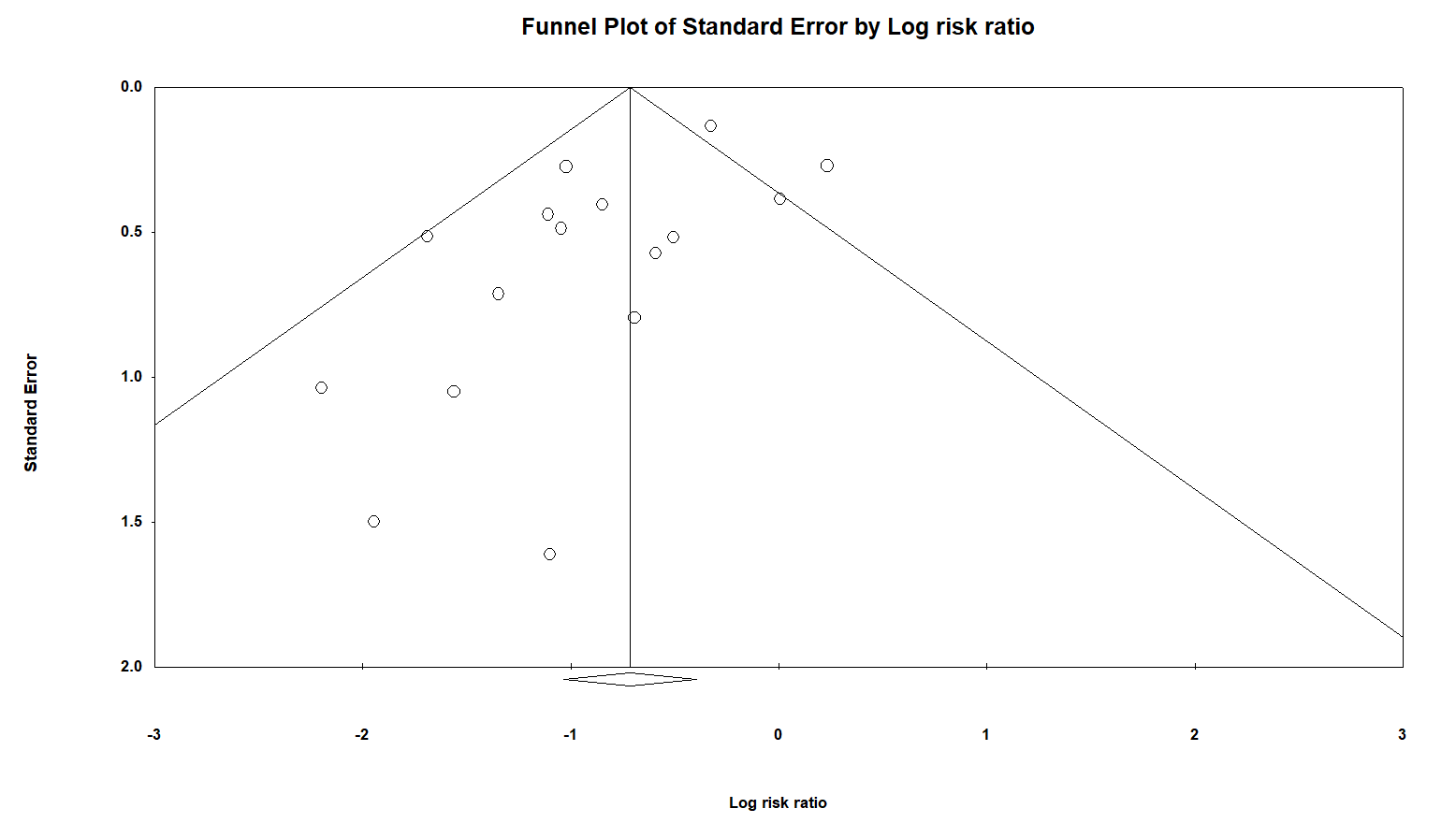


**Supplementary Figure S17** Infections Funnel Plot


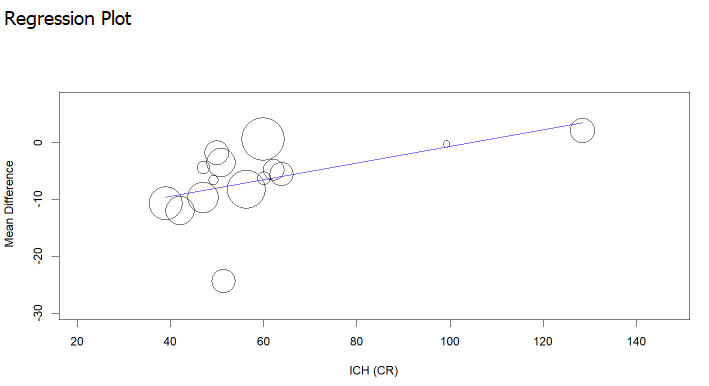


**Supplementary Figure S18** Regression plot of intracranial hemorrhage (ICH) volume and Hematoma evacuation rate for the Craniotomy (CR) group.


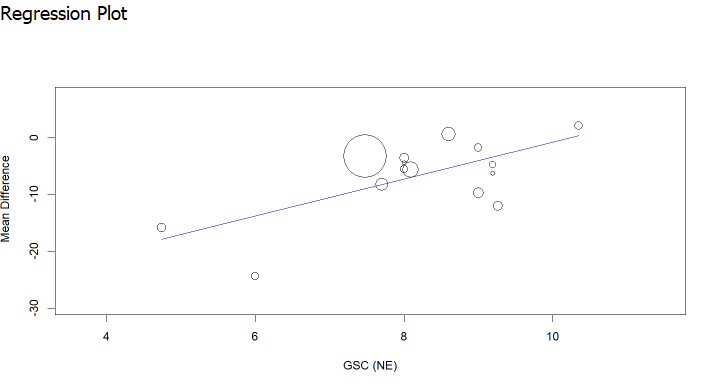


**Supplementary Figure S19** Regression plot of Glasgow coma Scale (GCS) score and Hematoma evacuation rate for Neuroendoscopy (NE) group


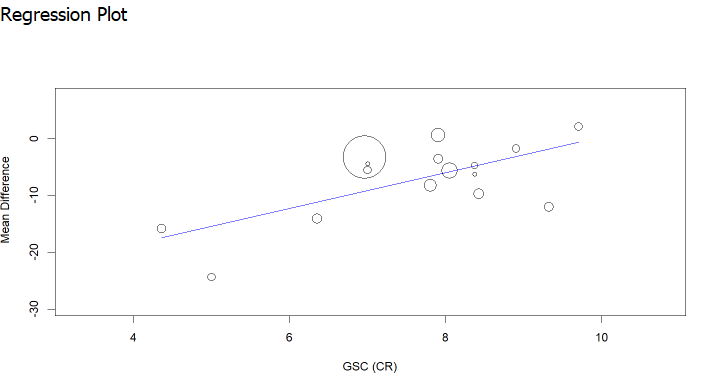


**Supplementary Figure S20** Regression plot of Glasgow coma Scale (GCS) score and Hematoma evacuation rate for Craniotomy (CR) group.


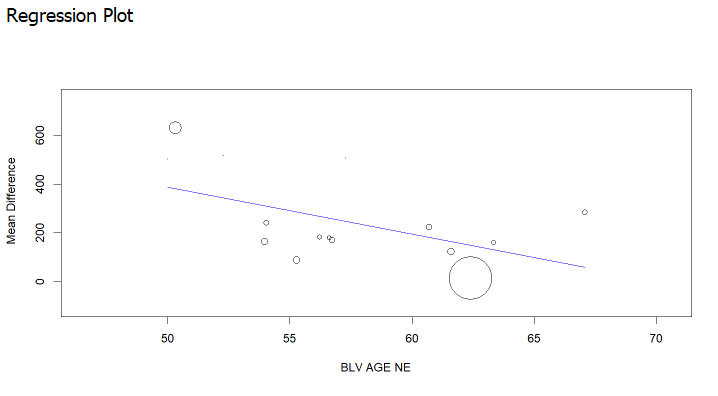


**Supplementary Figure S21** Regression plot of mean age and blood loss for Neuroendoscopy (NE) group


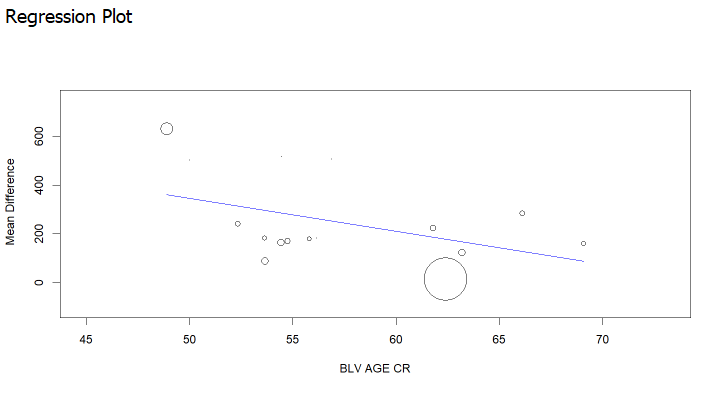


**Supplementary Figure S22** Regression of mean age and blood loss for the Craniotomy (CR) group.
